# Probiotic Gut Microbiota Isolate Interacts with Dendritic Cells via Glycosylated Heterotrimeric Pili

**DOI:** 10.1371/journal.pone.0151824

**Published:** 2016-03-17

**Authors:** Hanne L. P. Tytgat, Nienke H. van Teijlingen, Ruby May A. Sullan, François P. Douillard, Pia Rasinkangas, Marcel Messing, Justus Reunanen, Reetta Satokari, Jos Vanderleyden, Yves F. Dufrêne, Teunis B. H. Geijtenbeek, Willem M. de Vos, Sarah Lebeer

**Affiliations:** 1 Centre of Microbial and Plant Genetics, KU Leuven, Leuven, Belgium; 2 Department of Bioscience Engineering, Environmental Ecology & Applied Microbiology, University of Antwerp, Antwerp, Belgium; 3 Laboratory of Microbiology, Wageningen University, Wageningen, The Netherlands; 4 Academic Medical Center, Department of Experimental Immunology, University of Amsterdam, Amsterdam, The Netherlands; 5 Institute of Life Sciences, Université Catholique de Louvain, Louvain-la-Neuve, Belgium; 6 Department of Veterinary Biosciences, University of Helsinki, Helsinki, Finland; 7 Immunobiology Research Program and Department of Bacteriology and Immunology, University of Helsinki, Helsinki, Finland; University of Kansas Medical Center, UNITED STATES

## Abstract

Mapping of the microbial molecules underlying microbiota-host interactions is key to understand how microbiota preserve mucosal homeostasis. A pivotal family of such bacterial molecules are pili. Pili are proteinaceous cell wall appendages with a well-documented role in adhesion, whilst their role in immune interaction with the host is less established. Gram-positive pili are often posttranslationally modified by sortase-specific cleavage reactions and the formation of intramolecular peptide bonds. Here we report glycosylation as a new level of posttranslational modification of sortase-dependent pili of a beneficial microbiota species and its role in immune modulation. We focused on the SpaCBA pili of the model probiotic and beneficial human gut microbiota isolate *Lactobacillus rhamnosus* GG. A unique combination of molecular techniques, nanoscale mechanical and immunological approaches led to the identification of mannose and fucose residues on the SpaCBA pili. These glycans on the pili are recognized by human dendritic cells via the C-type lectin receptor DC-SIGN, a key carbohydrate-dependent immune tailoring pattern recognition receptor. This specific lectin-sugar interaction is moreover of functional importance and modulated the cytokine response of dendritic cells. This provides insight into the direct role bacterial glycoproteins can play in the immunomodulation of the host. Modification of the complex heterotrimeric pili of a model probiotic and microbiota isolate with mannose and fucose is of importance for the functional interaction with the host immune lectin receptor DC-SIGN on human dendritic cells. Our findings shed light on the yet underappreciated role of glycoconjugates in bacteria-host interactions.

## Introduction

Microbe-host interactions are vital for our wellbeing: the better the constitution and role of the human microbiota is known, the more it becomes apparent that microbiota homeostasis is health-related. An increasing number of studies suggest that dysbiosis of the microbiota can be linked to various disease states. However little is known about the molecular mechanisms governing host-microbiota interactions that modulate immune responses [1, 2].

Several big data projects, like the Human Microbiome Project (HMP) and the enterotype study, are mapping microbial diversity [3, 4]. Whilst these studies provide groundbreaking knowledge on the composition of the microbiota, it is concurrently important to delineate the key molecules underlying host-microbiota interactions. A better understanding of the structure of these molecules, their interaction mechanisms and their functional, immunogenic effects is primordial. An example of such relevant molecules are pili, which were also identified by the enterotype metagenomic study mining for functions relevant for intestinal microbes [3]. These cell wall appendages are known to be critical in microorganism colonization of the host epithelium, thus increasing their residence time [3, 5, 6]. Interestingly, in this metagenomics study, albeit *Escherichia coli* was reported as a low-abundant species, pili genes of *Escherichia* were recovered in abundance [3], illustrating the role of pili in the persistence of low-abundant microbiota species. Also other microbiota members have shown to be piliated, including several *Firmicutes* (e.g. *Lactobacillus* sp. [7] and *Streptococcus* sp. [8]), *Actinobacteria* (e.g. *Bifidobacterium* sp. [9, 10] and *Corynebacterium* sp. [11, 12]) [3, 4, 13].

In view of the pivotal adhesive role of these pili, the understanding of their structure and its relation to their functional role is key to fully grasp the complexity of microbe-host interactions. The structure of Gram-positive pili differs significantly from Gram-negative species. Most Gram-positive pili are covalent structures assembled from multiple protein subunits [14–16]. Generally they are composed of three subunits: a pilus backbone, a tip adhesin and a pilin decorating the pilus shaft [15, 16]. These subunits are posttranslationally assembled by sortase-mediated cleavage reactions and the formation of intramolecular peptide bonds. A housekeeping sortase covalently links the assembled pilus structure to the peptidoglycan backbone after recognition of an LPXTG motif [17, 18]. In contrast, Gram-negative are built by the non-covalent homopolymerization of a major pilin creating a pilus shaft to which additional pilins can be linked [16]. Currently little is known about the potential immunogenic role of both Gram-positive and Gram-negative pili. This is in clear contrast to the cell surface appendages for motility, i.e. flagella, which have a well-documented pro-inflammatory role in both Gram-negative and Gram-positive bacteria via interaction with Toll-like receptor (TLR) 5 [19]. Recently, it has been shown that innate and adaptive immunity interact to downregulate flagellar motility genes and in this way maintain mucosal homeostasis in the gut [19].

In the mucosal microenvironment immune cells are in close contact with the microbiota of the gut. Dendritic cells (DCs) are specialized sentinel cells of mucosal surfaces, which sense the microbiota through pattern-recognition receptors (PRRs). DCs express a plethora of intracellular and extracellular PRRs to deal with the diverse nature of microbes. Fine-tuning of innate signaling by these PRRs is important to orchestrate tailor-made responses to distinct microbes. The C-type lectin receptor (CLR) DC-SIGN efficiently binds mannose- and fucose-containing MAMPs (microbe-associated molecular patterns). Subsequent carbohydrate-specific signaling by DC-SIGN can modulate TLR-mediated responses and tailor adaptive immune responses [20–22]. Notably, DC-SIGN induces carbohydrate-specific signaling and is thought to play an important role in the tailoring adaptive immunity to specific pathogens [20–22]. However, it is unclear whether DC-SIGN interacts with carbohydrates expressed by the microbiota and how this affects immunity.

Here, we chose the beneficial microbiota isolate and model probiotic strain *L*. *rhamnosus* GG as a model as it produces the Gram-positive sortase-dependent heterotrimeric pili [23, 24]. Previous immuno-EM images observations showed that these bacterial cells are covered with 10 to 50 appendages of ~1 μm in length [7], i.e. the SpaCBA pili. Structural studies indicated that the pilus backbone is formed by SpaA and SpaC is the large tip adhesin. SpaB stops polymerization of the heterotrimeric pili and is mainly found at the pilus anchoring point, although some leaky expression across the pilus shaft is observed [25]. Also in *L*. *rhamnosus* GG, these SpaCBA pili are important adhesive molecules and key modulators of colonization and persistence in mucosa-associated niches [7, 26–28]. Earlier atomic force microscopy (AFM) studies have shown that this adhesion function is mediated via a zipper-like mechanism in which (the broad specificity of) the tip adhesin SpaC plays a key role [29, 30]. The location of SpaC on the tip facilitates the initial contact of *L*. *rhamnosus* GG with host cells, after which SpaC molecules dispersed along the pilus shaft establish the more intimate contact [29]. From an immunological perspective, their role is less unambiguous: pili have been shown to interact with macrophages [31], impact on TLR-2 signaling [26, 32] and their presence or absence modulates several pro- and anti-inflammatory cytokines [27, 31, 32], but these immunomodulatory functions could not be uncoupled from the adhesive functions promoting close contact with host immune cells.

Here we report on the glycosylation of SpaCBA pili of *L*. *rhamnosus* GG with mannose and fucose, and on the functional importance of this glycosylation. In particular we established that the glycans present on these pili are required for the interaction between *L*. *rhamnosus* GG and DCs via DC-SIGN. Given the complex trimeric structure of these pili, a multidisciplinary approach was used to describe the glycosylation combining nanomechanical, molecular, glycobiological and immunological techniques. This enabled us to present this report on the glycosylation of these peculiar bacterial sortase-dependent pili. Furthermore our data strongly suggest that these glycans are crucial for interaction with DCs and modulate immunity via DC-SIGN. Moreover, this study provides unique insights in the role of glycosylated cell wall molecules of an isolate of the human gut in the interaction with the host immune system, a factor that has been underappreciated.

## Material and Methods

### Bacterial strains and culture conditions

*L*. *rhamnosus* GG strains were grown at 37°C in MRS broth (Difco) in non-shaking conditions. In some cases, bacterial cells, dissolved in PBS, were heat killed (60°C, 15 min).

### Atomic force microscopy

AFM experiments were performed on live *L*. *rhamnosus* GG bacteria using the general procedure [33–35]. Two lectins were used, i.e. the fucose-specific *Aleuria aurantia* lectin (AAL, Vector Laboratories) and the *Hippeastrum* hybrid agglutinin (HHA) to detect mannose residues (kindly provided by Prof. Els Van Damme, UGhent). Wild type *L*. *rhamnosus* GG (ATCC 53103) and a pili-deficient Δ*spaCBA*::Tc^R^ mutant (CMPG5357) [27] were analysed, following single centrifugation at 4000 *g* for 2 min.

AFM tips were functionalized with lectins using ~6 nm long PEG-benzaldehyde linkers [36]. Cantilevers were first washed with chloroform and ethanol, placed in an UV-ozone cleaner for 15 min, and immersed overnight in 5.6 M ethanolamine hydrochloride (in DMSO) to generate amino groups on the tip surface. The cantilevers then reacted with PEG linkers carrying benzaldehyde groups on their free-tangling end for 2 hours, were afterwards rinsed with chloroform and immersed further in 1% citric acid solution for 10 min. After rinsing with Milli-Q water, the cantilevers were covered with 200 μL PBS solution containing 0.2 mg/mL lectins, to which 2 μL of a 1 M NaCNBH_3_ solution was added. After 50 min of incubation, 5 μL of a 1 M ethanolamine hydrochloride solution (pH 9.5) was added to block unreacted aldehyde groups (10 min). Finally, the cantilevers were washed with PBS and stored in the same buffer with added 1% sodium azide (NaN_3_), until use (within 7 days).

AFM measurements were performed at 25°C in PBS buffer (pH 7.4) supplemented with 1 mM CaCl_2_ and 1 mM MgCl_2_ using a Nanoscope VIII Multimode AFM (Bruker Corporation) and oxide sharpened microfabricated Si_3_N_4_ cantilevers (Microlevers, Veeco Metrology Group). The spring constants of the cantilevers were typically in the range of 0.02–0.04 N/m, as determined by the thermal noise method [37]. Cells were filtered and immobilized onto 1.2 μm diameter isopore polycarbonate membrane (Millipore). The membrane filter was gently rinsed 3 times with PBS, carefully cut into a ~1x1 cm^2^ square and attached to a steel sample puck using a double-sided adhesive and mounted onto the AFM liquid cell. Unmodified cantilevers were first used to image and localize individual *L*. *rhamnosus* GG cells. The unmodified cantilevers were then replaced by the lectin-functionalized tip. Force mapping was performed by collecting a 32x32 array of force-distance curves on a 300–500 nm^2^ localized area of the cell and the two-dimensional adhesion maps were reconstructed using the custom analysis described previously [38]. All force curves were recorded at ~100 ms contact time, with a maximum applied force of 250 pN and 2000 nm/s approach and retraction speeds, unless specified otherwise.

### Immunogold electron microscopy

Immunogold electron microscopy was done as described earlier with minor modifications [7, 39]. Briefly, the strains were grown overnight in MRS broth and washed gently twice with PBS. Freshly prepared formvar-carbon coated copper grids were incubated on top of cell suspension drops for 30 min at room temperature. The grids were then washed with 0.02 M glycine in PBS and blocked with 1% w/v BSA in PBS for 15 min. In the first labeling step, the grids were incubated with rabbit SpaA antiserum diluted 1:120 in blocking solution, followed by washing with 0.1% BSA in PBS. To detect the SpaA antibodies that bound to the samples, the grids were incubated on drops of protein A conjugated to 5 nm gold particles for 20 min. Protein A-gold was diluted 1:50 in blocking solution. The grids were washed with PBS, fixed with 1% v/v gluteraldehyde and then washed with water. To detect fucose, a second labeling was done using biotinylated AAL (Vector Laboratories), diluted 1:10 in blocking solution. After incubation on top of the lectin drops, the grids were washed with 0.1% w/v BSA in PBS and incubated with streptavidin-conjugated 10 nm gold particles (Sigma-Aldrich) diluted 1:5 in blocking solution. After washing with PBS, the grids were fixed with glutaraldehyde and washed with water prior to staining with a 1.8% methylcellulose-0.4% uranyl acetate mixture on ice. All washing steps in the protocol were performed several times. The grids were analysed using a JEM-1400 transmission electron microscope (JEOL).

### Purification of the SpaCBA pili of *L*. *rhamnosus* GG

*L*. *rhamnosus* GG cells (approx. 10^11^ CFU/mL) were grown in an industrial whey permeate based medium [40] and were a kind gift of Dr. Tuomas Salojarvi and Dr. Ross Crittenden (Valio Oy Finland). Cells were concentrated by microfiltration, resulting in retainment of the pili (unpublished observation). Approximately 500 mL of this concentrate was centrifuged for 30 min at 20 000 *g* in an Avanti J-26 XPI high-performance centrifuge (Beckman Coulter), which resulted in the shearing of the pili [30]. The supernatant (i.e. Pili Raw Material or PRM) was lyophilised (Heto FD 2.5, Heto Lab Equipment) and the residue dissolved in 1/25 of its original volume in PBS. The resulting solution was filtered twice, first through a 0.45 μm Millex GS syringe-driven filter (Millipore) and then using a 0.22 μm Millex HA syringe-driven filter (Millipore). 3 mL aliquots of the filtrate were run through a 120 mL Superdex 200 gel-filtration column (GE Healthcare) on an ÄKTApurifier 10 system (GE Healthcare) using PBS as the eluent, at a speed of 1 mL/min. Previous experiments had shown that the pili elute in the first protein peak. Twenty-four 1 mL fractions of the eluate were collected per run in a 96-deepwell plate. The registration of the absorbance at 280 nm was used to identify the fractions with high pili content.

Two pools were made: Pool A, containing the six fractions of each run nearest to the top of the absorbance peak, and Pool B, containing the six fractions of each run following those of Pool A (S3 Fig). Thus resulting in pure pili samples, with pool A having a higher molecular weight and purity (21 μg/mL) compared to pool B samples (9 μg/mL). Both pools were desalted by changing the buffer from PBS to 50 mM (NH_4_)_2_CO_3_/NH_4_HCO_3_ buffer, pH 8.0 using Zeba^™^ Spin desalting columns, 40K MWCO (Pierce Biotechnology). The resulting solutions were lyophilised (Heto FD 2.5) and the residues dissolved in 1/25 of their original volume of PBS and stored at -20°C. The purity of the pili preparations was assessed by Coomassie blue staining (S3 Fig), dot blot and probing of the blotted samples with a specific SpaC monoclonal antibody (kind gift of MicroDish BV and Podiceps BV) (results not shown). The purified pili samples (PRM, B and A) were aliquoted in small amounts, stored at -20°C and thawed prior to use.

### Purification of the *L*. *rhamnosus* GG Msp1 protein

Native Msp1 was purified as described earlier [41] using a combination of cationic exchange and ConA lectin affinity chromatography. Briefly, *L*. *rhamnosus* GG was grown for 24 h in AOAC medium. The supernatant of the culture (after centrifugation at 6 000 *g* for 20 min) was loaded on a SP Sepharose HighPerformance column (GE Healthcare), equilibrated with 60 mM lactate buffer (pH 4.0). The column was eluted using lactate buffer containing a NaCl gradient (100–1000 mM). The presence of Msp1 was verified using SDS-PAGE and the positive fractions were spin concentrated with Vivaspin filters with a MW cut off of 10 000 kDa (Sartorius Stedim biotech Gmbh). Msp1 was further purified from these positive fractions using a Hi-trap ConA 4B prepacked column (GE Healthcare).

### SpaC antiserum production

A recombinant SpaC *L*. *rhamnosus* GG protein (YP_003170190.1) covering amino acids 36 to 867 was produced by Genscript and was optimized for codon usage in *E*. *coli*. The synthetic gene (sequence and adaptations in S1 Methods) was cloned into the pET28b+ vector (Novagen) by using 5’*Nco*I and 3’*Xho*I for expression as a C-terminal His_6_ fusion protein in *E*. *coli*. Protein purification and rabbit immunization were done as described previously [42].

### Isolation of cell wall-associated proteins

Cell wall-associated proteins were isolated as described by Kankainen *et al*. [7] from wild type *L*. *rhamnosus* GG, the Δ*spaCBA*::Tc^R^ mutant lacking pili (CMPG5357) and the Δ*welE*::Tc^R^ mutant (CMPG5351) [35] on which the SpaCBA pili are overexposed due to the absence of the thick exopolysaccharides layer surrounding the cells. The obtained fractions were then separated on NuPAGE^®^ Novex^®^ 3–8% Tris-Acetate gels (Life Technologies), which are specifically designed to separate high molecular weight proteins. Western blotted samples were probed with SpaC antiserum, the mannose-specific lectin HHA (kind gift of Prof. Els Van Damme, UGhent) and the fucose-specific lectin AAL (Vector Laboratories). This assay was repeated in triplicate.

### Periodic Acid Schiff glycostain

Purified pili fractions were separated by SDS-PAGE on commercial NuPAGE^®^ Novex^®^ 3–8% Tris-Acetate gels (Life technologies). Glycostaining was performed using the commercial Periodic Acid Schiff base stain Pro-Q^®^ Emerald 488 (Molecular Probes^®^) following the manufacturer’s guidelines. Poststaining of the PAS stained gel with Sypro^®^ Ruby stain (Molecular Probes^®^) enabled visualization of the protein content of the sample. The gels were scanned using a Typhoon 9400 laser scanner (GE Healthcare). Purified Msp1 was used as a positive control.

The pili samples were also separated on Acrylamide Bis-Acrylamide gels (Amresco). The protein content of these samples was visualized using Sypro^®^ stain and Silver stain (SilverQuest^™^, Life Technologies). Glycosylation of the samples was tested using the Pro-Q^®^ Emerald 488 kit. Sypro^®^ and PAS stained gels were scanned with a Typhoon 9400 laser scanner. All assays were performed at least in triplicate for all purified pili fractions to verify the data.

### Lectin and antibody probing of Western blots

Purified pili samples and cell wall-associated proteins were separated by SDS-PAGE on Acrylamide Bis-Acrylamide gels (Amresco) and commercial NuPAGE^®^ Novex^®^ 3–8% Tris-Acetate gels. These gels were electroblotted to PVDF membranes (Life technologies) as previously described [41] and using the here mentioned tweaks. The blots were blocked with 0.5% polyvinylalcohol (PVA, Sigma-Aldrich^®^) to reduce background noise [43], which was dissolved in TBST (20 mM Tris-HCl, 500 mM NaCl, 0.1% Tween 20, pH 7.5) supplemented with 1 mM CaCl_2_ and 1 mM MgCl_2_ as cofactors for the lectins. All washing steps were performed using this TBST buffer with CaCl_2_ and MgCl_2_. The blots were probed with a plethora of eight digoxigenin-labeled lectins, including the mannose-specific *Galanthus nivalis* agglutinin (GNA) and HHA, the GlcNAc specific lectins UDA (*Urtica dioica* agglutinin), Nictaba and DSL (*Datura stramonium* lectin) (cf. S4 Fig) (kindly provided by Prof. Els J.M. Van Damme, UGhent), as described [44].

Both types of gels (Tris-Acetate gels and Acrylamide Bis-Acrylamide gels) were Western blotted and probed with SpaC antiserum to visualize their pili content [7]. Each purified pili fraction was tested as described at least in triplicate.

### Enzyme-linked lectin assay (ELLA)

Purified pili (5 μg/mL, sample A) were dissolved in 50 mM sodium carbonate buffer (pH 9.6) and coated overnight onto ELISA plates (X50 Immunolon 4HBX plates, Thermo Scientific) at 4°C. For the lectin probing using the mannose-specific lectin HHA, mannan was used as a positive control (5 μg/mL). In the case of the fucose-specific lectin AAL, Lewis X was coated as a positive control (5 μg/mL) (Lectinity). Wells only containing 50 mM sodium carbonate buffer were used as negative controls for the assay. The next day the wells were washed trice with PBS, preceding the blockage of the wells with 0.5% PVA dissolved in TBS [43] for 2-3h at 37°C. The wells were then washed with PBS prior to the addition of the digoxigenin-labeled lectins dissolved in TBS + 0.5% PVA (1h, 37°C). Unbound lectins were removed by washing the wells with PBST (PBS + 0.1% Tween 20). Then the secondary antibody, anti-digoxigenin (Roche) [44] was added to the cells for 1 hour at 37°C. Prior to development, the wells were washed with PBST. 4-nitrophenyl phosphate disodium salt (1mg/mL, Sigma-Aldrich^®^) dissolved in substrate buffer (carbonate bicarbonate buffer, pH 9.6) was added to each well. Color was allowed to develop for 15–30 min at room temperature and the absorbance was measured at 405 nm.

### DC-SIGN-Fc ELISA

High-binding flat-bottom 96-well ELISA plates were coated overnight with 50 mL of 50 mg/mL ligand, i.e. pili sample B, dissolved in 0.2 M coating buffer at room temperature. Mannan (1 mg/mL, Sigma-Aldrich^®^) was coated as a positive control, whilst plain coating buffer served as negative control. After two washes with TSM (20 mM TrisHCl, 150 mM NaCl, 1 mM CaCl_2_, 2 mM MgCl_2_) the wells were blocked with 1% BSA (Sigma-Aldrich^®^) for 30 minutes at 37°C. DC-SIGN-Fc fragments were 15 minutes preincubated with blocking agents in TSM: the D1 DC-SIGN specific antibody (20 mg/mL) and calcium chelator EGTA (10 mM). After washing, the plate was incubated with 50 μL of DC-SIGN-Fc (with block) for 2 hours at room temperature on a shaking platform. The wells were washed with TSM with 0.1% Tween 20 (TSMT), prior to the addition of goat anti-human Fc-PO (Sigma-Aldrich^®^) dissolved in TSMT (30 minutes). After extensive washing with TSMT, the ELISA could be developed using a 10 mL substrate solution of 0.1 M NaAc, TMB and H_2_O_2_. 15 minutes later this reaction was stopped by the addition of 0.8 M H_2_SO_4_. Experimental read-out was performed at OD_450_.

### Interaction between pili-coated beads, bacteria, Raji(DC-SIGN) cell lines and monocyte-derived DCs

Beads were coated with pili as described [45]. In short, streptavidin was covalently coupled to fluorescent beads (TransFluoSpheres, 1 μm, Alexa 488/Alexa647) and further coated by subsequent incubation with biotinylated Swine-anti-rabbit-antibody (20 μg/mL, 2h at 37°C, Sigma-Aldrich^®^), SpaC antiserum (5 μg/mL, overnight 4°C) and purified pili (sample B, overnight 4°C).

Bacteria were labeled with FITC (Sigma-Aldrich^®^) by mixing 50 μL of 1 mg/mL FITC (Sigma) in DMSO with 500 μL 2x10^8^ bacteria in PBS, incubated for 1 hour at RT and washed extensively.

Raji and Raji-DC-SIGN cells [46] were cultured in RPMI (Gibco) supplemented with 10% FCS, 2500 U/mL penicillin, 2500 μg/mL streptomycin, 100 mM L-Glutamine and passaged twice a week.

CD14+ monocytes from healthy individuals were isolated from buffy coats that were obtained from Sanquin Blood Bank in Amsterdam, The Netherlands. The study was approved by the Institutional Review Board (IRB) of Sanquin Blood Bank (IRB reference number NVT0257) and is in accordance with the ethical guidelines of the Academic Medical Center and the Declaration of Helsinki. All blood donors gave informed consent and remained anonymous throughout all experimental procedures. Isolated CD14+ monocytes were stimulated for 6 days with IL-4 (500 U/mL, Biosource) and GM-CSF (800 U/mL, Invitrogen) to differentiate into immature DCs as described previously [47, 48].

5x10^4^ moDCs, Raji or Raji-DC-SIGN cells were preincubated for 30 min at 37°C with TSA (TSM supplemented with 0.5% Albumin), DC-SIGN specific antibody D1 (20 μg/mL), Lewis X (20 μg/mL) or EGTA (5 mM, Sigma-Aldrich^®^), respectively. Next, cells were incubated for 1 hour with FITC-labeled bacteria at MOI 20, washed extensively, fixed in 4% vol/vol PFA, and anlyzed on FACSCanto^™^ II (BD Biosciences).

With the fluorescent beads, 5x10^4^ moDCs, Raji or Raji-DC-SIGN cells were preincubated for 30 min at 37°C with TSA, DC-SIGN specific antibody D1 (20 μg/mL), Lewis X (20 μg/mL), or EGTA (5 mM), respectively. Next, cells were incubated for 30 minutes with 3 μL beads, washed extensively, fixed in 4% PFA, and analyzed on FACSCanto^™^ II (BD, Biosciences).

### Cytokine induction

1x10^5^ moDCs were pre-incubated with medium or DC-SIGN specific antibody D1 (10 ug/mL) for 1 hour and thereafter stimulated with 250 pg/mL pili sample B. mRNA was isolated after 6 hours using the lysis buffer from the mRNA Capture kit (Roche). cDNA was made with a cDNA synthesis kit (Promega). Amplification and real-time quantification was performed by PCR with SYBR Green according to manufacturer’s guidelines for the ABI 7500 Fast PCR detection system (Applied biosciences).

### Data analysis

Data analysis was performed using GraphPad Prism^®^ 6. Significant differences between two values were calculated using paired t-tests and the significance level was set at p <0.05.

## Results

### Microscopic techniques reveal SpaCBA pili glycosylation with mannose and fucose

AFM [49, 50] was previously used to assess the nanomechanical properties of the SpaCBA pili [29] and surface polysaccharides of *L*. *rhamnosus* GG [34]. Here AFM was applied to evaluate the glycosylation profile of these pili on living *L*. *rhamnosus* GG cells using AFM tips functionalized with mannose- and fucose-specific lectins (*Hippeastrum* hybrid agglutinin (HHA), and *Aleuria aurantia* lectin (AAL), respectively). Lectins were chosen in view of abundant sugars in the gut mucus and earlier knowledge on recurring sugars in microbiota members, like the mannose sugars present on the major secreted proteins of *L*. *rhamnosus* GG itself [41] and fucose on the typical gut symbiont *Bacteroides fragilis* [51, 52].

**Fig 1:**
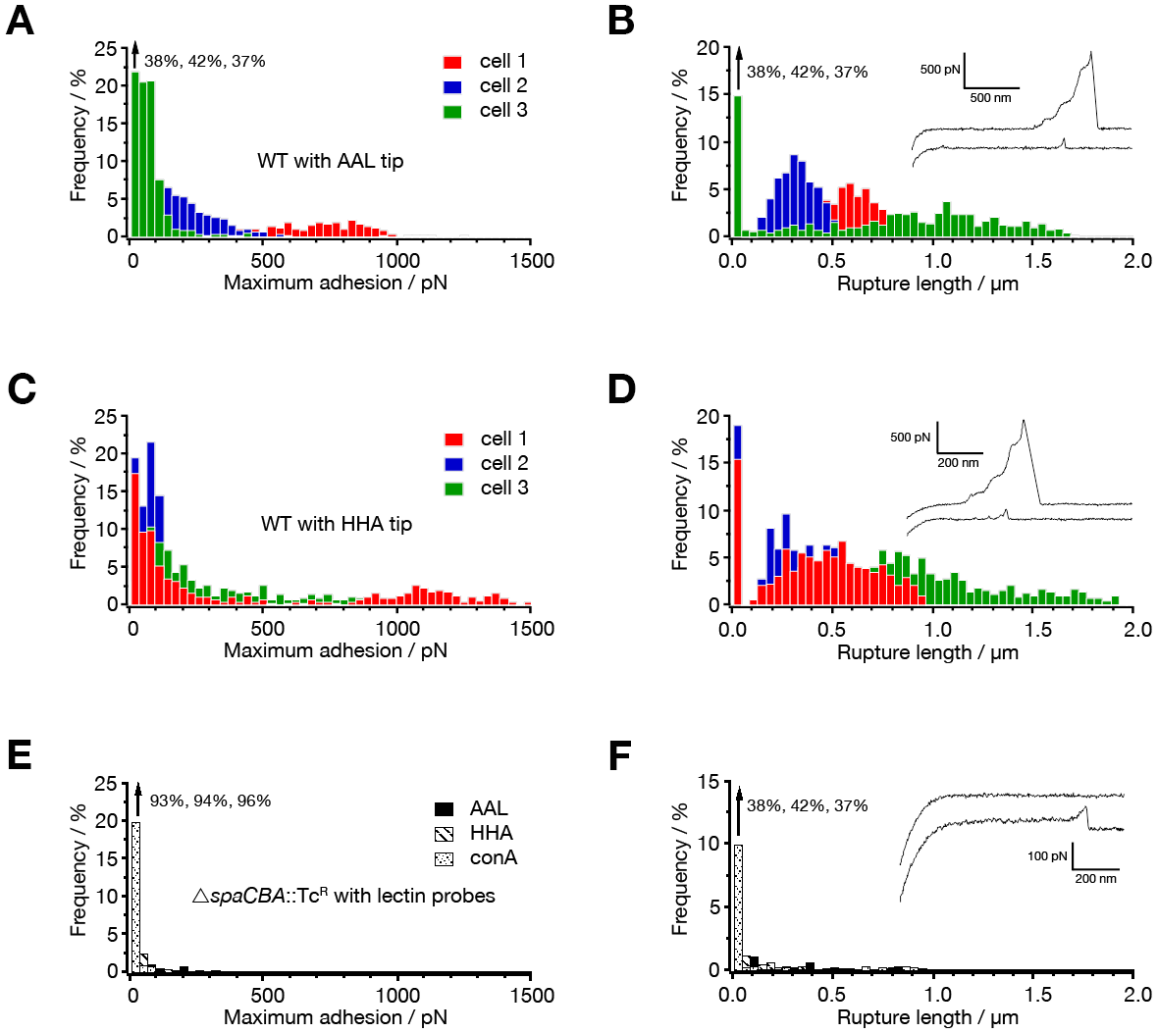
Probing of fucose and mannose residues on *L*. *rhamnosus* GG pili using AFM. Fig 1A and 1C depict the adhesion forces and Fig 1B and 1D the rupture length histograms (n = 1024) obtained in buffer, from the interaction between *L*. *rhamnosus* GG wild type and fucose- and mannose-binding lectin probes (AAL and HHA resp.). In Fig 1E and 1F the force data for the interaction of a pili-deficient Δ*spaCBA*::Tc^R^ mutant (CMPG5357) with the two lectin probes are displayed. Insets show representative retraction force curves.

Wild type *L*. *rhamnosus* GG cells adhered specifically to both lectin probes, as exemplified by the adhesion force curves (Fig 1A–1D). As a control, these force curves were shown to be absent when the Δ*spaCBA*::Tc^R^ mutant (CMPG5357) lacking the SpaCBA pili [27] was probed (Fig 1E and 1F). Closer examination of force curves from wild type revealed two distinct adhesive behaviors. A large fraction of the adhesive curves featured single adhesion peaks with moderate forces, mostly in the 50–200 pN range (lower curve in the insets in Fig 1B and 1D), which can be ascribed to specific lectin-sugar interactions [34]. A significant fraction of the curves (from 10% to 40% depending on the cell and on the probe, with HHA giving a higher fraction (~39%) as compared to AAL (~26%)) represent much larger adhesion forces, in the range of 500–1500 pN, and extended rupture lengths (500–2000 nm) (upper curve in the insets in Fig 1B and 1D). Consistent with earlier AFM experiments on pili [29, 38], these force peaks showed linear shapes and constant force steps, reflecting the nanospring behavior of the *L*. *rhamnosus* GG pilus. This finding strongly suggests that the two lectin probes specifically bind to the *L*. *rhamnosus* GG SpaCBA pili, thus indicating that they are likely to be modified with strongly associated fucose and mannose residues. The force signatures were moreover reproducibly observed with the lectin tips, which would not be possible if sugars are loosely associated or adsorbed. Supporting these observations, pili-less cells (Δ*spaCBA*::Tc^R^) showed no significant binding events (Fig 1E) and a complete lack of nanospring profiles (Fig 1F).

**Fig 2:**
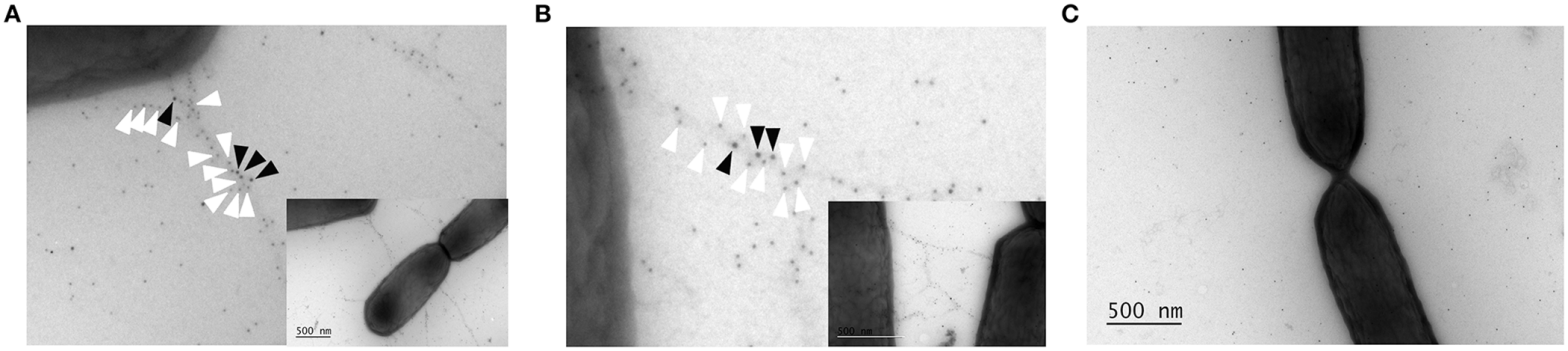
Immunogold labeling reveals colocalization of SpaA and fucose on SpaCBA pili. Immunoelectron microscopy double labeling of *L*. *rhamnosus* GG cells (A and B) and the Δ*spaCBA*::Tc^R^ mutant (CMPG5357) (C) with SpaA antiserum and the fucose-specific *Aleuria aurantia* lectin (AAL). Detection of SpaA and AAL was done using 5 nm (white arrows) and 10 nm gold particles (black arrows) respectively. The scale bar represents 500 nm. Original overall pictures are shown as insets of A and B.

As fucosylated glycoproteins are until now only documented in members of the *Bacteroidetes* phylum of the microbiota, the fucosylation of these SpaCBA pili was further substantiated by immunogold electron microscopy. Coincubation of wild type *L*. *rhamnosus* GG cells with SpaA antibody and the fucose-specific AAL lectin resulted in the colocalization of both on the SpaCBA pili (Fig 2A and 2B). Similar experiments using the *spaCBA*::Tc^R^ mutant lacking pili did not result in the binding of either of the particles (Fig 2C).

### Lectin blotting confirms SpaCBA pili glycosylation

**Fig 3:**
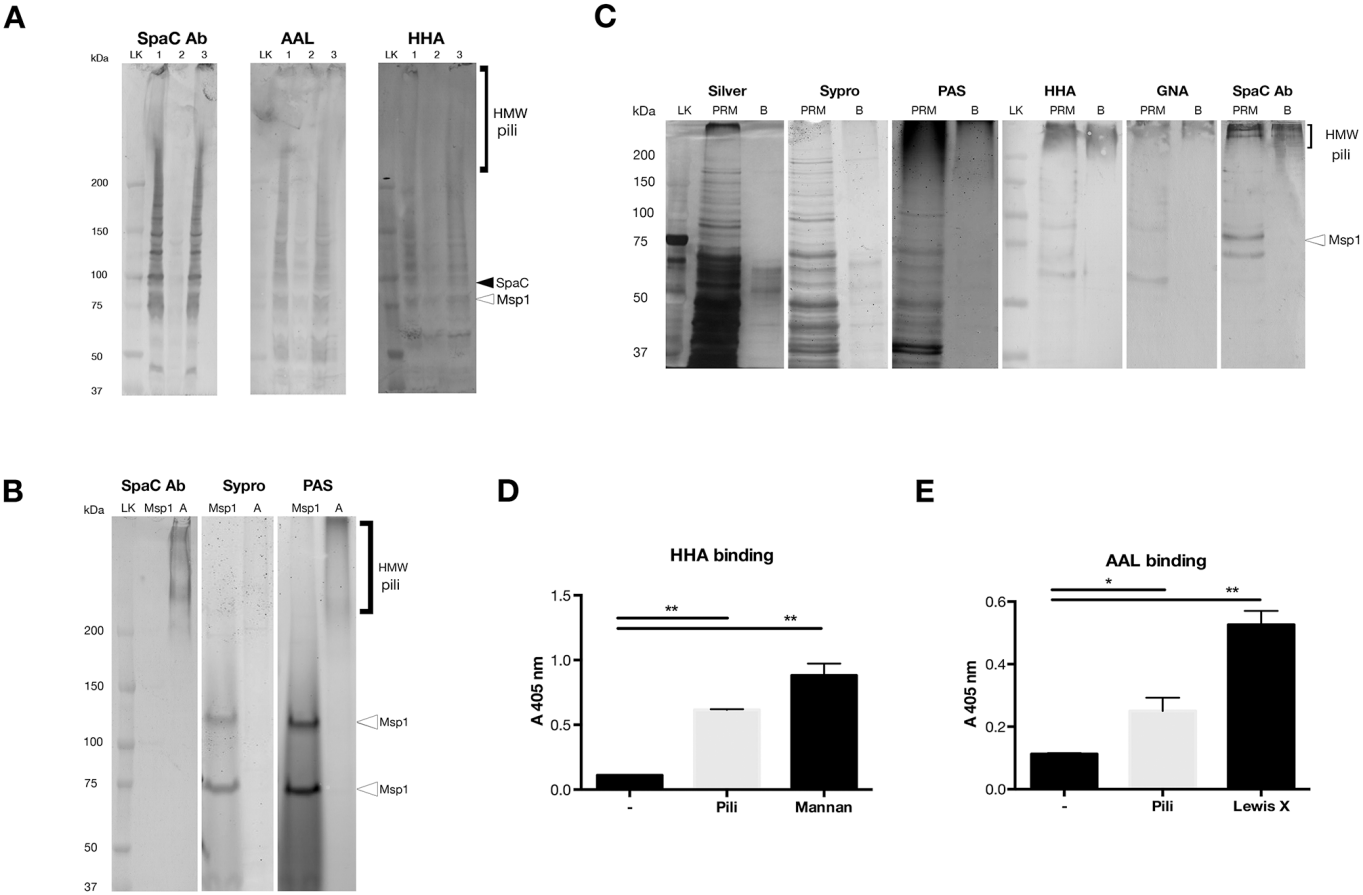
SpaCBA pili are glycosylated with mannose and fucose. **(A) SpaCBA pili are glycosylated on *L*. *rhamnosus* GG cells—**Cell wall-associated proteins of *L*. *rhamnosus* GG wild type (1), the pilus-deficient Δ*spaCBA*::Tc^R^ mutant (CMPG5357, 2) and the Δ*welE*::Tc^R^ mutant on which the pili are overexposed (CMPG5351, 3), were probed with mannose- and fucose-specific lectins (HHA and AAL resp.). Pili content was visualized by probing with SpaC antiserum (SpaC, black arrow and HMW: high molecular weight pili). Interference of the Msp1 glycoprotein was ruled out (open arrow). Blots and gels were performed in triplicate. (LK = Precision Plus Protein^™^ Kaleidoscope^™^ Standard, Bio-Rad) **(B) Purified pili are glycosylated—**SDS-PAGE separated pili (pool A) were stained with PAS glycostain and Sypro^®^ to visualize their protein content. Pili content was shown by probing of Western blotted samples with SpaC antiserum (HMW: high molecular weight pili). Purified Msp1 (open arrow) was used as a positive control. Representative gels are shown, experiment was carried out in triplicate. (LK = Precision Plus Protein^™^ Kaleidoscope^™^ Standard, Bio-Rad) **(C) SpaCBA pili bind mannose-specific lectins—**Purified pili fractions (PRM and pili pool B) were subjected to PAS glycostaining and both Sypro^®^ and Silver stain to visualize their protein content. Absence of 75 kDa signals on PAS and lectin blots rule out the interference Msp1 (cf. open arrow). Western blotted samples were probed with SpaC antiserum (HMW: high molecular weight pili) and the mannose-specific lectins HHA and GNA, visualizing the pili content of the samples and the presence of mannose, respectively. Representative gels and blots of in triplicate-repeated experiment. (LK = Precision Plus Protein^™^ Kaleidoscope^™^ Standard, Bio-Rad) **(D&E) Mannose- and fucose-specific lectins bind SpaCBA pili—**Binding of lectins to plate-coated pili was measured to ELISA. Wells coated with coating buffer served as a negative control. Mannan and Lewis X were coated as a positive control for the mannose-specific HHA (*Hippeastrum* hybrid, D) and fucose-specific AAL (*Aleuria aurantia*, E) lectins, respectively. Error bars represent standard deviations of three independent experiments (paired t-test, p < 0.05)

Cell wall-associated proteins were subsequently isolated from wild type *L*. *rhamnosus* GG, the pilus deficient Δ*spaCBA*::Tc^R^ strain (CMPG5357) and the Δ*welE*::Tc^R^ mutant (CMPG5351), which lacks the thick exopolysaccharides layer, resulting in an overexposure of the SpaCBA pili [35]. Western blotted samples were probed with SpaC antiserum in parallel with lectin probing with AAL and HHA, resp. (Fig 3A). Both wild type *L*. *rhamnosus* GG and Δ*welE*::Tc^R^ cell wall-associated proteins did bind these lectins (Fig 3A). The presence of Msp1 (open arrow in Fig 3A) and other glycoproteins account for residual signals on the lectin blots for the Δ*spaCBA*::Tc^R^ mutant. These results further corroborate our earlier microscopic findings on the presence of fucose and mannose on the SpaCBA pili of *L*. *rhamnosus* GG.

### Purified SpaCBA pili carry mannose and fucose residues

To exclude interference of other sugar conjugates on the cell surface with the microscopic and Western Blot-based lectin probing experiments described above, heterotrimeric SpaCBA pili were also purified from wild type *L*. *rhamnosus* GG cells to confirm their glycosylation status. In view of the complexity of Gram-positive pili, the purification entailed a complex multi-step procedure including shearing off pili by high-speed centrifugation followed by gel filtration, aiming at three different fractions with increasing purity (PRM, B and A fraction, S3 Fig). These purified pili fractions all reacted with the Periodic Acid Schiff glycostain (PAS), especially in the high molecular weight range (i.e. >200 kDa) (fraction A, Fig 3B; PRM and B fraction, Fig 3C), which is typically the region in which the SpaCBA pili can be found after SDS-PAGE [7]. This is corroborated by a Western blot probed with SpaC antiserum (Fig 3B and 3C). Protein content was visualized using Sypro^®^ and silver stain (Fig 3B and 3C). As a control, purified Msp1 was subjected to the same procedure (Fig 3B, cf. open arrows), resulting in a typical ca. 75 kDa band [41] and a higher glycostained band (complex or oligomer of Msp1). The absence of this ca. 75 kDa band in samples B and A and its very low abundance in the PRM fraction rules out interference of this glycoprotein with the pili glycosylation signal (Fig 3B and 3C).

All purified pili fractions were subsequently probed with a range of lectins with different sugar specificities (mannose, fucose, N-acetylglucosamine, galactose, glucose, N-acetylgalactosamine) (cf. Material & Methods) [44]. The mannose-specific lectins GNA and HHA elicited a strong binding with the high molecular weight fraction of the pili samples (Fig 3C), in addition to fucose-specific lectin AAL (results not shown). The other lectins tested did not show any binding in the high-molecular weight area of the Western blotted samples (S4 Fig). Enzyme-linked lectin assays (ELLA) was then performed to confirm the Western blot results, as these assays are less prone to aspecific binding of lectins. ELLA of the purified pili probed with the mannose-specific lectin HHA (Fig 3D) and fucose-specific lectin AAL (Fig 3E) confirmed their mannosylation and fucosylation. These experiments thus delineate fucose and mannose monomers as the two most important pili-modifying sugars in *L*. *rhamnosus* GG.

### Purified SpaCBA pili bind recombinant DC-SIGN

**Fig 4:**
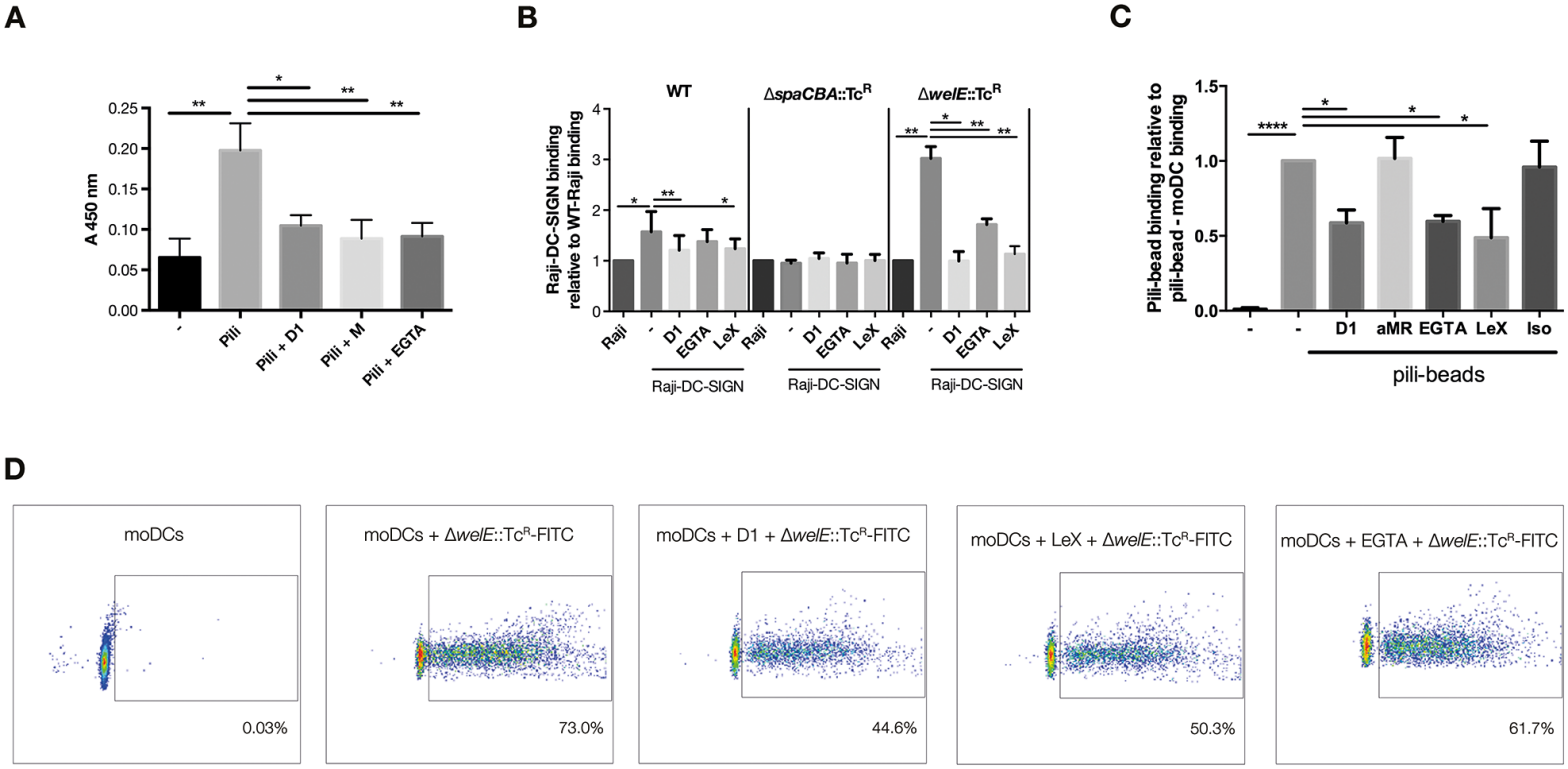
**Glycosylated SpaCBA pili of *L*. *rhamnosus* GG interact with DCs via DC-SIGN (A) DC-SIGN-Fc interact specifically with SpaCBA pili.—**DC-SIGN binding to plate-coated pili was measured by ELISA. Wells coated with coating buffer and mannan (data not shown) served as negative and positive control, respectively. DC-SIGN-Fc was pre-exposed to TSM buffer, DC-SIGN specific antibodies (D1), mannan (M) or EGTA, respectively, before incubation on pili-coated wells. Error bars represent standard deviations of three independent experiments (paired t-test, p < 0.05). **(B) Piliated *L*. *rhamnosus* GG strains interact Raji-DC-SIGN cell line**—Untransfected and DC-SIGN expressing Raji cells were incubated with FITC-labeled wild type *L*. *rhamnosus* GG, Δ*spaCBA*::Tc^R^ and Δ*welE*::Tc^R^. Binding was determined by flow cytometry. DC-SIGN specific antibodies (D1), a Lewis X carbodydrate structure (LeX) and EGTA were used to determine specificity of DC-SIGN binding to *L*. *rhamnosus* GG variants. Binding is expressed relative to binding of FITC-labeled bacteria to untransfected Raji cells. Error bars represent standard deviations of three independent experiments (paired t-test, p < 0.05). **(C) Purified pili bind DCs via DC-SIGN—**Monocyte-derived DCs (moDCs) were incubated with pili-coated beads and binding was determined by flow cytometry. DC-SIGN binding specificity was assessed using DC-SIGN specific antibodies (D1), a Lewis X carbodydrate structure, and EGTA. Binding is expressed relative to unblocked binding of pili-beads to moDCs (N = 3, paired t- test, p < 0.05). **(D) *L*. *rhamnosus* GG with overexposed pili recognition by DCs—**Representative FACS plots of Δ*welE*::Tc^R^ binding to moDCs that were preincubated with TSA buffer (no block), DC-SIGN specific antibodies (D1), a Lewis X carbodydrate structure (LeX) and EGTA. On the x-axis: binding of FITC-labeled bacteria to moDCs as measured in the FL1 channel of the FACS. On the y-axis: forward scatter.

Since HHA and AAL are plant and fungus-derived lectins resp., used as molecular tools rather than reflecting possible hosts of *L*. *rhamnosus* GG, we subsequently investigated the functional ramifications of the glycosylation of the pili of the beneficial gut microbe *L*. *rhamnosus* GG with human immune lectins relevant for the intestinal niche. Since the CLR DC-SIGN has been shown to strongly interact with both mannosylated and fucosylated structures on pathogens [48], we investigated the interaction of DC-SIGN with pili using recombinant DC-SIGN in an ELLA-based set-up [53]. Notably, purified pili interacted with DC-SIGN and this binding could be inhibited by a specific antibody against DC-SIGN (Fig 4A) [48]. Mannan also inhibited pili binding to DC-SIGN, further supporting a role of this CLR binding to the SpaCBA pili. Blockage of binding with EGTA further corroborates the sugar-lectin nature of the interaction between the pili and DC-SIGN, as Ca^2+^ is an important cofactor enabling DC-SIGN binding [48].

### DC-SIGN expressing cell line binds both purified SpaCBA pili and piliated bacteria

To validate the DC-SIGN interaction in a more physiological context, we then studied the interaction between the pili and cellular DC-SIGN [54]. Interestingly, DC-SIGN expressing Raji cells interacted strongly (60%) with pili-coated beads in contrast to untransfected Raji cells (S1 Fig). DC-SIGN specific antibodies as well as the fucosylated DC-SIGN ligand Lewis X (LeX) [55] inhibited pili binding to DC-SIGN. The interactions of pili with DC-SIGN were shown to be Ca^2+^ dependent as EGTA abrogated the interaction.

Next, we investigated whether pili expressed by different bacteria are recognized by DC-SIGN. Wild type *L*. *rhamnosus* GG significantly interacted with the DC-SIGN expressing Raji cells, compared to untransfected Raji cells (Fig 4B). EPS-lacking Δ*welE*::Tc^R^ mutant bacteria were even recognized more strongly by DC-SIGN, which is most probably due to the higher accessibility of pili. In contrast, the non-piliated Δ*spaCBA*::Tc^R^ bacterial cells could not specifically interact with the DC-SIGN expressing cell line, indicating the importance of the pili for the interaction between *L*. *rhamnosus* GG and the CLR DC-SIGN. Binding of wild type and Δ*welE*::Tc^R^ bacteria could be inhibited by antibodies against DC-SIGN, Lewis X and EGTA, further supporting the important role of pili in DC-SIGN interactions.

### Glycosylated SpaCBA pili mediate the interaction between *L*. *rhamnosus* GG and primary DCs via DC-SIGN

To further investigate the interaction with DC-SIGN, we subsequently studied whether the glycosylated SpaCBA pili on *L*. *rhamnosus* GG are recognized by primary DCs. Interestingly, pili immobilized on beads clearly interacted with primary DCs and this interaction could be partially blocked by DC-SIGN specific antibodies, EGTA, and LeX, whereas neither the isotype antibody nor antibodies directed against the Mannose Receptor (MR) inhibited the interaction (Fig 4C). These data suggest that DC-SIGN on DCs binds the SpaCBA pili of *L*. *rhamnosus* GG. Subsequently, the role of glycosylated SpaCBA pili in the interaction between fluorescent FITC-labeled live bacteria and DCs was investigated. Similar to wild type, bacteria with overexposed pili (Δ*welE*::Tc^R^) interacted with DCs and the interaction was partially blocked by specific antibodies against DC-SIGN, LeX and to a minor extent with EGTA (S2 Fig, Fig 4D). Notably, DC interaction with non-piliated Δ*spaCBA*::Tc^R^ bacteria was not dependent on DC-SIGN, as this interaction was not susceptible to blocking by specific ligands (S2 Fig). These data strongly indicate that the glycosylated SpaCBA pili expressed by *L*. *rhamnosus* GG are involved in the interaction with DC-SIGN on DCs.

### SpaCBA pili elicit a cytokine response in DCs

**Fig 5:**
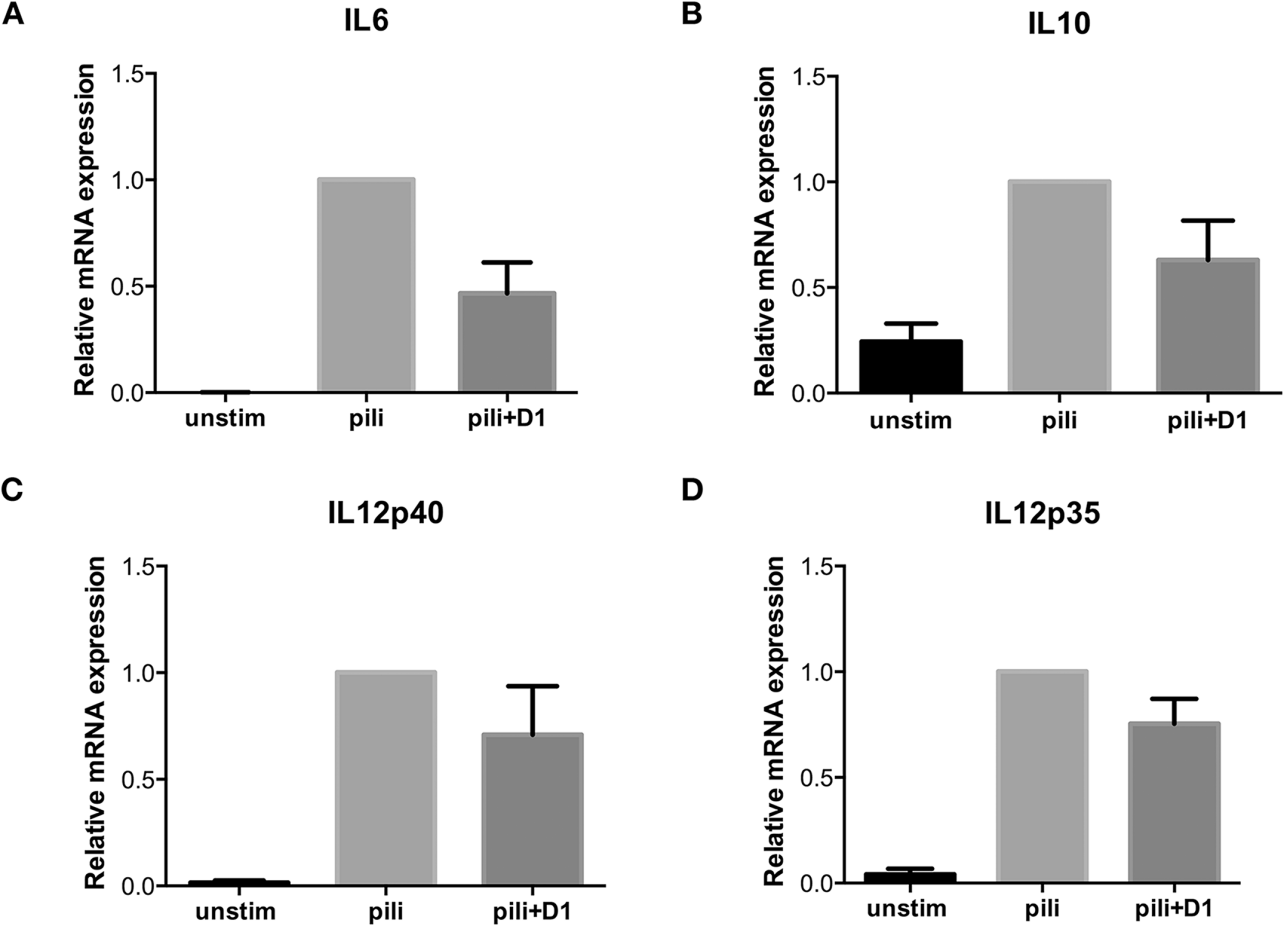
Purified pili induce cytokine expression by DCs. Monocyte-derived DCs were stimulated with purified pili (sample B) for 6 hours after which IL-6, IL-10, IL-12p40 and IL-12p35 mRNA expression was determined by RT-PCR. DC-SIGN specific antibodies (D1) were used to assess DC-SIGN specificity of cytokine induction. Cytokine expression values represent relative cytokine expression to moDCs incubated with pili (N = 4).

DCs are important in inducing adaptive immunity and DC-SIGN has been shown to be involved in the tailoring of adaptive immunity to pathogens [20, 21]. Here we investigated whether SpaCBA glycosylated pili affect DC function and induce immunity. Notably, incubation of immature DCs with purified SpaCBA glycosylated pili led to an induction of different cytokines i.e. IL-6, IL-10, IL-12p40 and IL-12p35 (Fig 5). Next, we investigated whether DC-SIGN was involved in the immune response since DC-SIGN is known to be a modulator of cytokine production induced through TLRs [20, 56, 57]. Hereto, DCs were exposed to purified pili in the presence of antibodies against DC-SIGN. Interestingly, IL-6 and IL-10 expression, and to a lesser extent IL-12p40 and IL-12p35 expression were partially blocked by antibodies against DC-SIGN (Fig 5). These data strongly indicate that glycosylated pili are involved in inducing and modulating DC function via DC-SIGN.

## Discussion

Pili are crucial molecules in host-microbe interactions, which are increasingly thought to be of importance for the adhesive capacity of specific gut microbiota members [3]. A good understanding of their structure allows to further comprehend the molecular details of these specific interactions. Here, we report on the posttranslational modification of sortase-dependent multimeric structures in a beneficial Gram-positive bacterium, namely pilus glycosylation. Our findings corroborate recent findings by Morello *et al*. on the glycosylation of pili in *Streptococcus agalactiae* [58]. The fucosylated and mannosylated SpaCBA pili of *L*. *rhamnosus* GG interact with the CLR DC-SIGN on human DCs, thereby modulating adaptive immune responses. This work provides new insights on how beneficial bacteria and microbiota members can interact functionally with the immune system using glycosylated ligands.

Given the complex nature of these sortase-dependent pili, an innovative combination of complimentary techniques was needed to confirm their glycosylation. First evidence was derived from specifically designed AFM studies with lectin probes. Scanning of the cell surface of *L*. *rhamnosus* GG with lectin-modified AFM tips resulted in force curves reflecting the known nanospring and adhesion behavior of SpaCBA pili, corroborating their modification with mannose and fucose residues [29]. Colocalization of gold particles labeled with SpaA antibody and fucose-specific lectin enabled the visual confirmation of the presence of fucose-residues on the SpaCBA pili of *L*. *rhamnosus* GG. These microscopy-based results were further underpinned by antibody and lectin-probing experiments on Western blotted purified pili fractions and cell wall associated proteins of *L*. *rhamnosus* GG cells. These combined efforts resulted in the delineation of fucose and mannose as the most important sugars modifying the SpaCBA pili of *L*. *rhamnosus* GG. Both purified pili and (non-)piliated bacteria were used to rule out any interference of the complex purification process (e.g. contamination) and evaluate the importance of the glycosylation of the SpaCBA pili in the presence of other cell wall molecules.

Fucose and mannose are important sugars in the intestinal tract. For instance, an intimate interaction between *Bacteroides* microbiota members and host-derived fucose is well documented, whereby *Bacteroides* incorporates exogenous fucose into capsular polysaccharides and glycoproteins via dedicated biosynthetic pathway [52, 59, 60]. How fucose and mannose and other sugars are attached to sortase-dependent heterotrimeric pili of *L*. *rhamnosus* GG, and other commensal microbiota, remains to be further investigated. The complex heterotrimeric structure of the SpaCBA pili hampered our efforts to elucidate their glycosylation using state of the art mass spectrometry for the detection of glycans on glycoproteins. Yet, the fact that a similar nanospring behavior was observed with the AFM-tips modified with lectins as with the SpaC specific antiserum indicate that these SpaC subunits are the pilins decorated with sugar residues, but further research is needed to corroborate this hypothesis. Interestingly, it was suggested earlier that these SpaC pilins harbor a von Willebrand factor domain with weak homology to a fucose-binding lectin domain [7]. Previous AFM studies on the specificity and binding properties of SpaC indicate that this pilin has a broad specificity (towards sugars *and* proteins), resulting in zipper-like functioning, but also shows rapid dissociation rates [29]. The fucose-binding lectin-like domain of the pili might bind fucose, rendering a non-covalently bound fucosylation signal in our assays. This is however highly unlikely as the lectin AFM assays would not be reproducible if the sugars would only be adsorbed or loosely bound to the pili as they would detach and remain bound to the tip, making further detection impossible. The high consistency and reproducibility of all assays both using intact bacteria and purified SpaCBA pili and various washing steps in all procedures further corroborate this. Dedicated glycan and glycoprotein assays are needed to fully elucidate the biochemical character of the pili glycosylation.

We here present not only the specific glycosylation of complex heterotrimeric pili in beneficial bacteria, but also clues on the importance of its glycosylation for the establishment of specific interactions with important immune cells, namely DCs. On DCs, the CLR DC-SIGN specifically recognizes fucose- and mannose-MAMPs on microorganisms [46, 48]. Here we show that glycosylated SpaCBA pili of *L*. *rhamnosus* GG specifically interact with DC-SIGN on primary DCs. Notably, this interaction with DC-SIGN on DCs modulates adaptive immune responses. Although the interaction between DC-SIGN and the commensal microbiota is less explored, it is well documented that DC-SIGN binds different pathogens and thereby modulates DC-mediated immune responses [20, 22, 56]. Our data strongly indicate that glycosylated pili expressed by microbiota members such as *L*. *rhamnosus* GG are important in maintaining immune homeostasis via crosstalk with CLRs, like DC-SIGN.

The immunomodulatory effect of DC-SIGN signaling depends on the presented MAMP; mannose-structures enhance the expression of TLR-induced pro-inflammatory cytokines, while fucose-based ligands enhance IL-10 production but inhibit expression of IL-6 and IL-12. Here we show that purified heterotrimeric pili of a commensal microbiota isolate induce IL-6, IL-10, IL-12p40 and IL-12p35 expression in DCs and the induction of these cytokines was partially dependent on DC-SIGN. To what extent this influences adaptive immunity needs to be further addressed. The cytokine expression profile suggests that the mannose structures on the pili are involved in the DC-SIGN-induced immune responses. DC-SIGN binding to carbohydrates on pili might affect accessibility to and signaling via TLRs. Interestingly, pili have been shown to impact TLR-2 signaling [26, 32], which might be modulated by DC-SIGN.

Taken together we report on the glycosylation of the heterotrimeric adhesive pili of the gut microbiota isolate *L*. *rhamnosus* GG. Furthermore, we could show the importance of the fucosylation and mannosylation of the SpaCBA pili for the interaction with the CLR DC-SIGN on human DCs, thus generating insights in the importance of glycans in microbiota-host interactions and immunomodulation. This work provides new insights in the piliation of bacteria and aims to encourage the study of the potential impact of pili glycosylation in other important piliated microbiota members and bacteria in general. Our results corroborate the findings from the hallmark metagenomic study of the different enterotypes in the gut, where pili were shown to be of high importance for low-abundance species in the gut [3].

## Supporting Information

S1 Fig Interaction of pili-beads and Raji-DC-SIGN cell line. (DOCX)

S2 Fig CLR DC-SIGN recognizes glycans on *L*. *rhamnosus* GG SpaCBA pili. (DOCX)

S3 Fig Pooled fractions of purified pili result in samples A and B. (DOCX)

S4 Fig Screening of purified SpaCBA pili with GlcNAc-specific lectins. (DOCX)

S1 Methods Recombinant SpaC production in *E*. *coli*. (DOCX)
