# Supplementary material for: Probiotic Gut Microbiota Isolate Interacts with Dendritic Cells via Glycosylated Heterotrimeric Pili
Source: PLoS One. 2016 Mar 17;11(3):e0151824. doi: 10.1371/journal.pone.0151824 (PMC4795749; doi:10.1371/journal.pone.0151824)
Supplement: S1 Fig — (DOCX) [file pone.0151824.s001.docx]

**
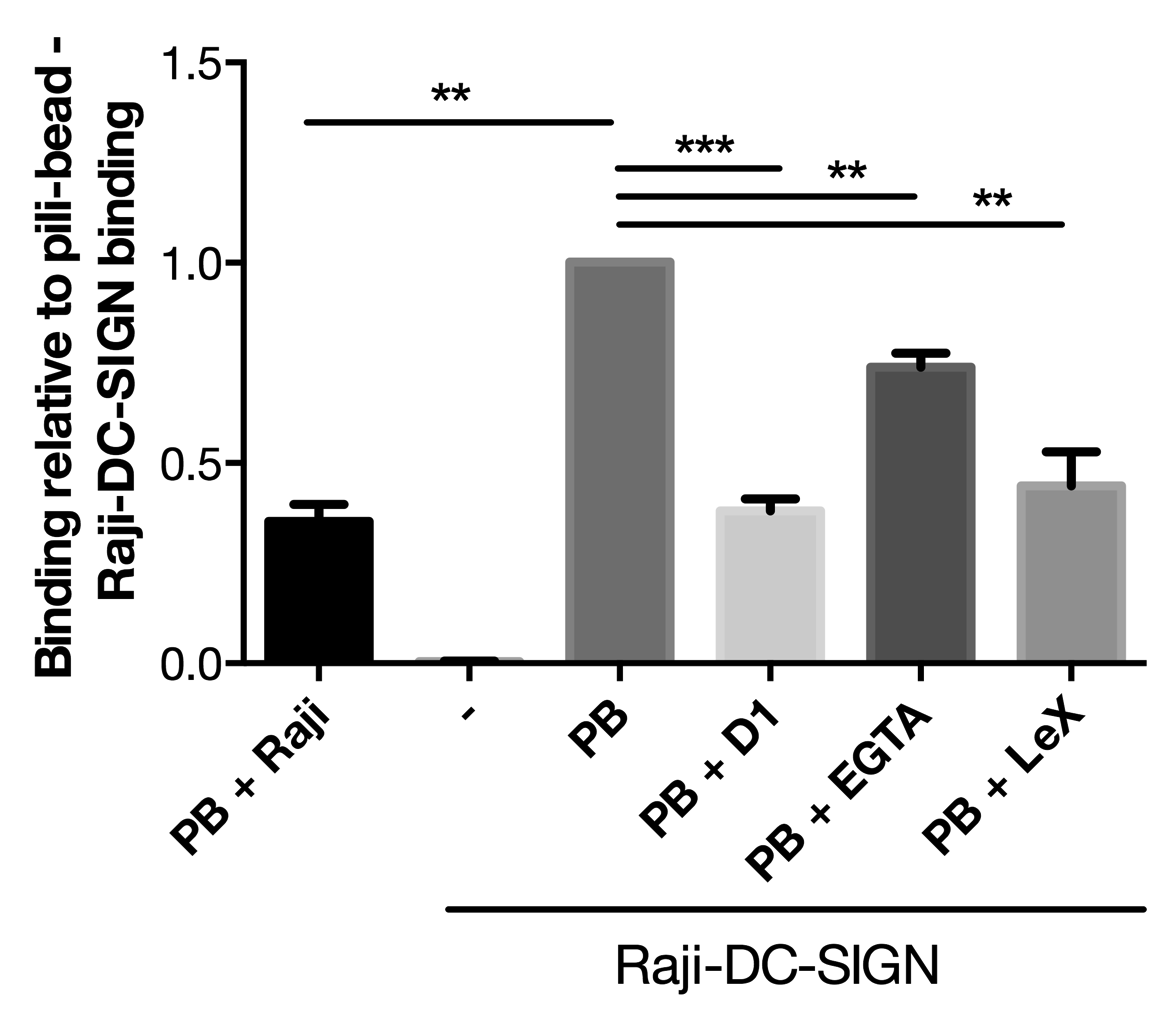
**

**S1 Fig - Interaction of pili-beads and Raji-DC-SIGN cell line -** Untransfected and DC-SIGN expressing Raji cells were incubated with pili-coated beads and binding was determined by flow cytometry. Antibodies against DC-SIGN (D1), a Lewis X carbohydrate structure (LeX), and EGTA were used to determine specificity of DC-SIGN binding. Binding is expressed relative to unblocked binding of pili-beads to Raji-DC-SIGN cells. Error bars represent standard deviations of three independent experiments. (p < 0.05)
