# Supplementary material for: Probiotic Gut Microbiota Isolate Interacts with Dendritic Cells via Glycosylated Heterotrimeric Pili
Source: PLoS One. 2016 Mar 17;11(3):e0151824. doi: 10.1371/journal.pone.0151824 (PMC4795749; doi:10.1371/journal.pone.0151824)
Supplement: S2 Fig — (DOCX) [file pone.0151824.s002.docx]

**
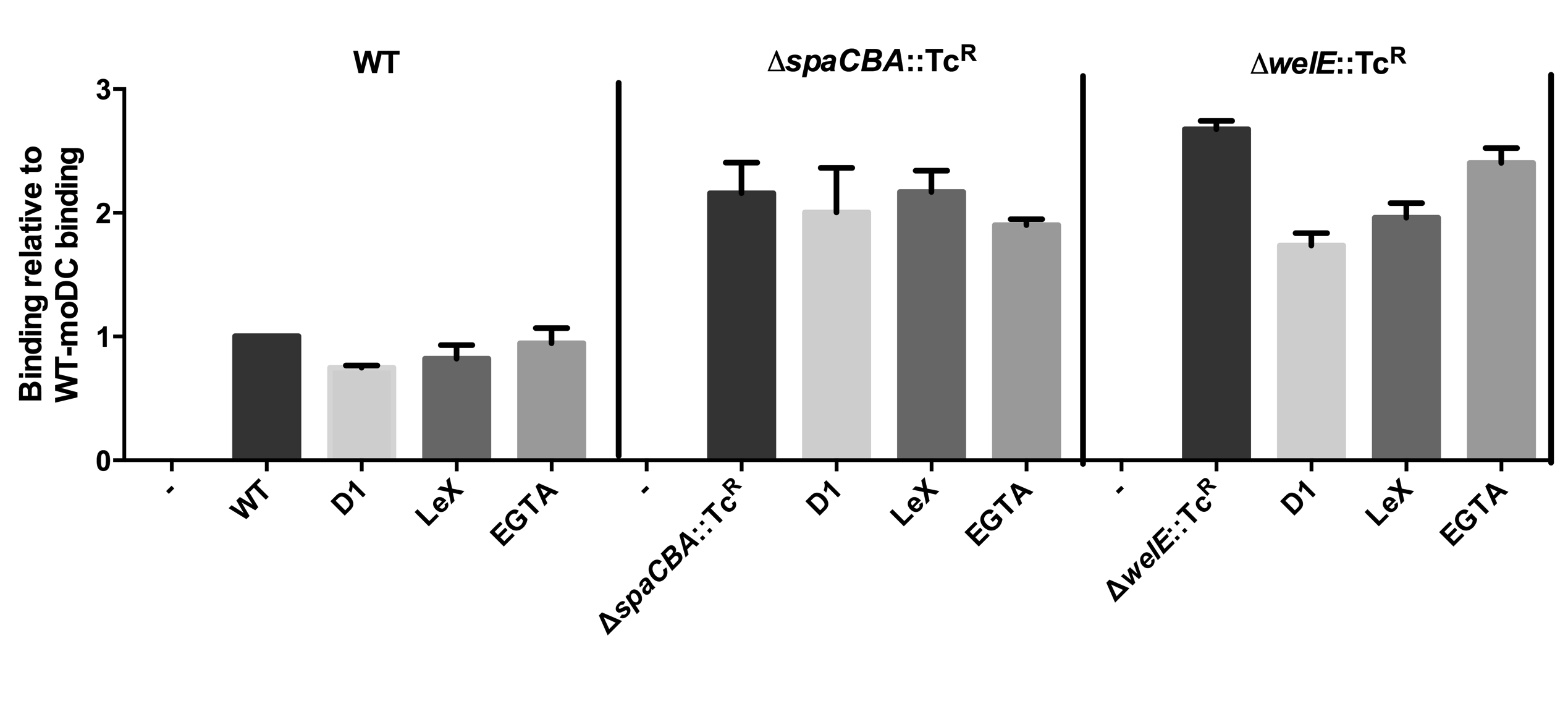
S2 Fig - CLR DC-SIGN recognizes glycans on *L. rhamnosus* GG SpaCBA pili -** FITC-labeled wild type *L. rhamnosus* GG, Δ*spaCBA*::Tc^R^, and Δ*welE*::Tc^R^ were incubated with moDCs and binding was determined by flow cytometry. Specificity of binding to DC-SIGN was determined using DC-SIGN specific antibodies (D1), a Lewis X carbodydrate structure (LeX) and EGTA. All values represent relative binding to the unblocked binding of wild type *L. rhamnosus* GG to moDCs. A representative experiment is depicted; equivalent observations were obtained in independent repetitions of the experiment.
