# Supplementary material for: Probiotic Gut Microbiota Isolate Interacts with Dendritic Cells via Glycosylated Heterotrimeric Pili
Source: PLoS One. 2016 Mar 17;11(3):e0151824. doi: 10.1371/journal.pone.0151824 (PMC4795749; doi:10.1371/journal.pone.0151824)
Supplement: S3 Fig — (DOCX) [file pone.0151824.s003.docx]

**
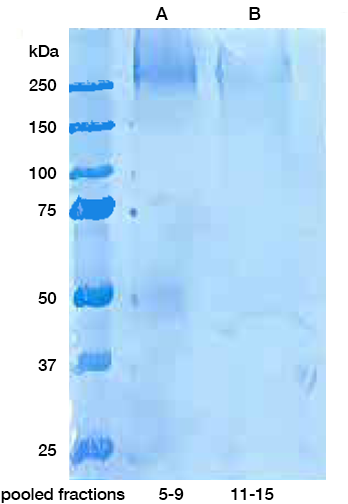
**

**S3 Fig - Pooled fractions of purified pili result in samples A and B**

A Coomassie Brilliant Blue stained gel illustrates protein content of the pili purified samples A and B. The six fractions with the highest absorbance were pooled to generate sample A, whilst the next six fractions, still containing pure pili, were pooled generating sample B.
