# Supplementary material for: Probiotic Gut Microbiota Isolate Interacts with Dendritic Cells via Glycosylated Heterotrimeric Pili
Source: PLoS One. 2016 Mar 17;11(3):e0151824. doi: 10.1371/journal.pone.0151824 (PMC4795749; doi:10.1371/journal.pone.0151824)
Supplement: S4 Fig — (DOCX) [file pone.0151824.s004.docx]

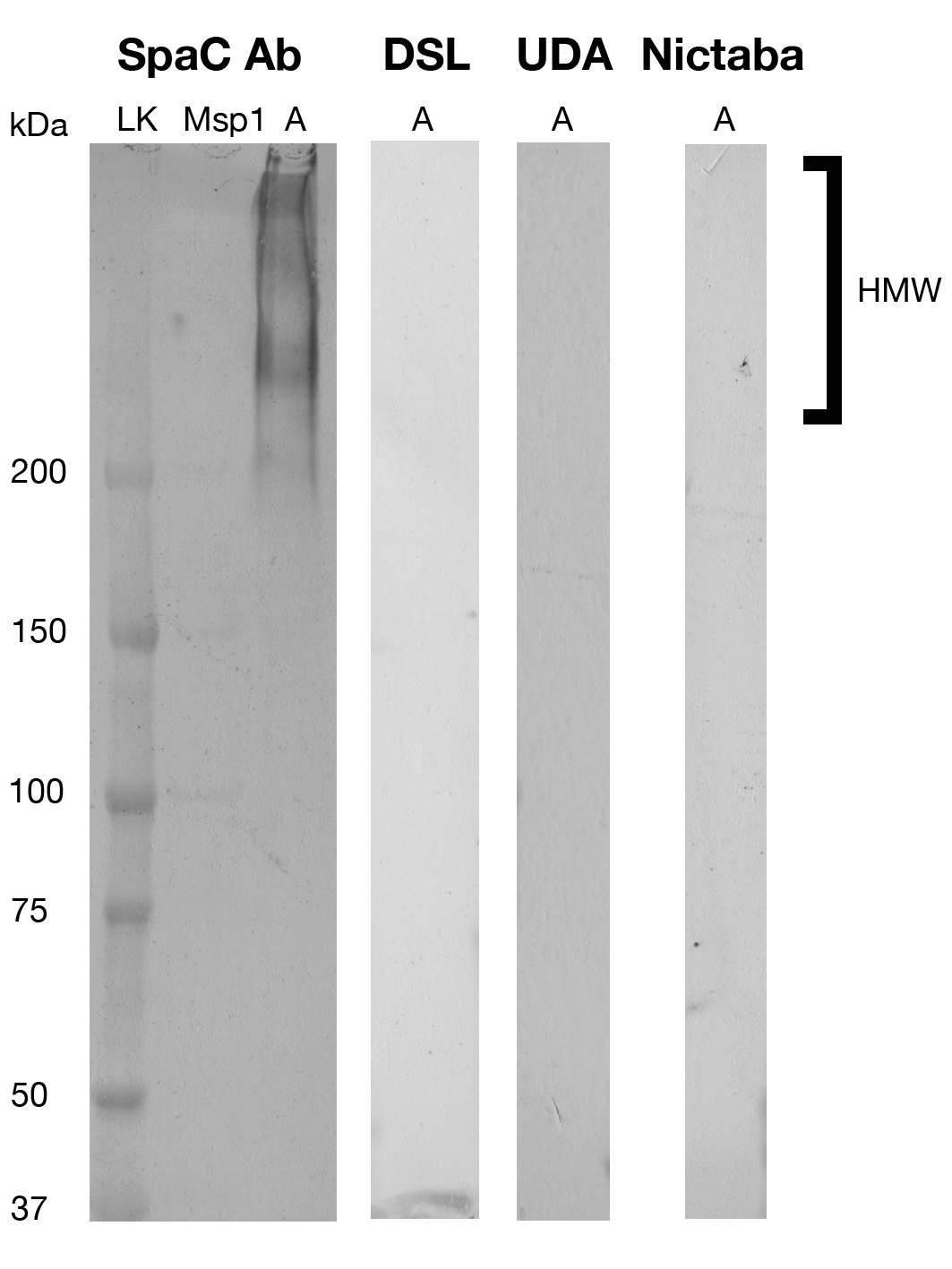


**S4 Fig – Screening of purified SpaCBA pili with GlcNAc-specific lectins**

Purified pili (sample A) separated by SDS-PAGE (Tris-Acetate gel, cf. M&M) were probed with a range of lectins specific for GlcNAc. These lectins could not bind to the SpaCBA pili. Experiment was performed in triplicate, representative blots are shown. (HMW: high molecular weight pili, DSL: *Datura stramonium* lectin, UDA: *Urtica dioica* agglutinin)
