# Supplementary material for: Probiotic Gut Microbiota Isolate Interacts with Dendritic Cells via Glycosylated Heterotrimeric Pili
Source: PLoS One. 2016 Mar 17;11(3):e0151824. doi: 10.1371/journal.pone.0151824 (PMC4795749; doi:10.1371/journal.pone.0151824)
Supplement: S1 Methods — (DOCX) [file pone.0151824.s005.docx]

**S1 Methods**: **Recombinant SpaC production in *E. coli***

The SpaC coding sequence (YP_003170190.1), ranging from amino acid 36 (threonine, codon bolded) to 867 (leucine, codon bolded) was optimized for codon usage in *E. coli* by Genscript and 5’ *Nco*I and 3’ *Xho*I restriction sites (underlined) were added to the sequence. The synthetic gene designed for the production of the histidine-tagged recombinant SpaC is depicted below, together with start codon (ATG for methionine, bold and underlined) and additional glycine codon in the 5´ end (in italics), and additional 3´ leucine and glutamate (bold and underlined) codons followed by the hexahistidine (His_6_, in italics) sequence and the stop codon (TGA).

**Codon optimized *spaC* sequence for heterologous protein expression in *E.coli:***

CC**ATG***GGC***ACC**GACAACATTCGTCCGACCTATCAAACCGATGCTAACGGCACCTATCCGACCAACTCGTGGCAAGTGACCGGCCAACAAAACGTTATTAATCAACGCGGCGGTGACCAGGTCTCAGGCTGGGATAACAATACCATCTGGAATGGTGACGCGACGGATACCACGAACTCGTATCTGAAATTTGGCGACCCGAACAATCCGGATTATCAGATTCGTAAATACGCCAAGGAAACCAATACGCCGGGCCTGTATGATGTGTACCTGAACGTTAAAGGTAATAAGCAGCAAAACGTTAAACCGGTCGACATCGTTCTGGTGGTGGATATGAGCGGTTCTATGGAAAGTAATCGTTGGGGTACCAACCGTGCAGGTGCTGTCCGTACCGGTGTGAAAAATTTTCTGACGAGCATTCAGAACGCGGGCCTGGGTAACTATGTTAATGTCGGCCTGATCGGTTTCAGCTCTCCGGGCTATATCGGCGGTAAATCAGGTTACATTTCGGTTAAACTGGGCAAGGCCGGTAATGCTAGCCAGCAACAGGCAATTAACGGTGCTCTGTCTCCGCGTTTTCAGGGCGGTACCTACACGCAAATCGGCCTGCGTCAGGGTAGTGCGATGCTGAATGCCGACACCTCCGGCAACAAAAAGATGATGATTCTGCTGACCGATGGTGTCCCGACGTTTTCAAATGAAGTGATTAACTCGGAATGGATCAATGGCACCCTGTATGGTACGAACTTCGGCAGTTCCCGCGATGAACCGGGTAATACCGCACGTCTGCGCTGGCCGTACACGGACTCATCGGGCCATTATATCTACGATACCTGGCCGGCGACGCTGGGTGAAGCGAAAATTGCCAAGGATAGTGGCAACGAAGTGCATGCACTGGGTATCCAGCTGGCTGATGACGATCACTATATGACCAAAGAAAAGATCCGTCAAAATATGCAGCTGATTACGAACTCACCGGACCTGTATGAAGACGCGGATTCGGCGGATGCCGTTGAAGCGTACCTGAACAATCAGGCCAAGGATATCATCAAGAACTTCAACACCGTGACGGACGGCACCATTACGGATCCGATCGGTACCCAATTCCAGTATGCAAACAATCAGGCTACCGTTACGAGCGTCGGCAAACAGACCGTTCCGGCAAGCGAACTGCCGTCTGCAGCCATTCAAGACGGCCAGCTGACCGTGAACCATATGAATCTGGGTCAAGATCAGGAAGTGCAAATCCACTATCAGGTTCGCATTAAAACCGAAGACGCCGGTTTTAAGCCGGATTTCTGGTACCAGATGAACGGCGAAACCCTGCTGACGCCGAAAGCAGGTGCAGCTGCGGTGGATTTTGGTATCCCGAGCGGTCGTGCACCGGCTACCACGGTGTATGTTCAAAAACAGTGGCGTCAACTGAGCAATCAGTCTCTGCCGGATACCCTGAACGTCACGGTGCAGCGTAAAGTTGCGGACGGCTCCCTGGATCCGAATTGGCAACAGACCCTGGTCCTGAAAAAGGCGGATAACTGGAAAGCCTCTTTTACCGCGCCGGCCTATAACAATCAAGGCCAGAGTTTCTCCTACGTCGTGAAAAGTGAAGACGCGTCCGGTATCGATCTGAGCTCTTTCATTAGTTCCCAGAATATGGATCAACAGACCGCCACGCTGACCCTGACGAACCAACAGTATGGCTTTCAATTCCAGAAAAAGACCACGGACGGTACCGATCTGAGCGCAGACCAACTGAAAGCTATGCAGTTTAATCTGACCCAGTACAGTGATAACTCCTTCCAACAGGCGAGCAAGACGAATGCCATTACCTCTACGGATCTGCAGGCACTGGCTCCGGGCTATTACGGTATCCAGGAAGCCGCAGCTCCGACCGGCTATCAGCTGGATGGTACCACGTACCTGTTTCAGCTGACCAGCGACGGCCAATGGCAGTATCATGGTACCAAAGATAACGTTACGTCAGGCTCGGTCATTAATGGTCAACAGACCCTGAACCCGGTGGGCGACAAATCTGACGATTTCACCGTTACGGGTGATCACCAACAGATTCTGACCCTGACGAAATATGACGAACCGAAGCCGAGCATGACCCTGCGCGTTATCAAACAGGATAATCAATCTCAGTACCTGGCAGGTGCAGCATTTACCCTGCAGCCGAGTGCAGGTGAAGCTGAAACCATTACGTCATCGGCAACCTCCGAGGGTCAGGCATTCGCAACGAAACTGGTGGCAGACGGTACCTATACGATGAGTGAAACCAAGGCTCCGGATGGCTACCAGTCCAACCCGGCGAAAATTGCCATCCAGGTCGCAACCACGGGCAAGGAAGCTACCGTGACGATTGATGGTGAAGCCCTGAAACCGGGCGAATCAAAGAATGGTTATACCCTGGCAATTGATGGCTCGACCATCACGCTGCAAGCGATTAACCAGCCGCTGGCAATCCTGCCGCACACCGGCGGTCAAGGTTACCAGCGTCTGCTG**CTCGAG***CACCACCACCACCACCAC*TGA

**Alignment of the *spaC* sequence (amino acids 36 to 867) optimized for codon usage in *E. coli* (SpaC-c) and the *L. rhamnosus* GG *spaC* sequence depicting the optimized nucleotides:**

10 20 30 40 50 60

SpaC-c ACCGACAACATTCGTCCGACCTATCAAACCGATGCTAACGGCACCTATCCGACCAACTCG

:: :: :::::::: :: :::::::::::::::::::: :: ::::::::::: :: :::

SpaC ACTGATAACATTCGCCCAACCTATCAAACCGATGCTAATGGTACCTATCCGACAAATTCG

110 120 130 140 150 160

70 80 90 100 110 120

SpaC-c TGGCAAGTGACCGGCCAACAAAACGTTATTAATCAACGCGGCGGTGACCAGGTCTCAGGC

::::: :: :: :: :::::::: :: :: :::::::: ::::: :: :: :: :::::

SpaC TGGCAGGTCACGGGACAACAAAATGTAATCAATCAACGTGGCGGGGATCAAGTTTCAGGG

170 180 190 200 210 220

130 140 150 160 170 180

SpaC-c TGGGATAACAATACCATCTGGAATGGTGACGCGACGGATACCACGAACTCGTATCTGAAA

:::::::: ::::: :: ::::::::::: ::::: :::::::::::::: :: ::::::

SpaC TGGGATAATAATACAATATGGAATGGTGATGCGACTGATACCACGAACTCTTACCTGAAA

230 240 250 260 270 280

190 200 210 220 230 240

SpaC-c TTTGGCGACCCGAACAATCCGGATTATCAGATTCGTAAATACGCCAAGGAAACCAATACG

::::: ::::: :: :::::::::::::::::::: ::::: :: :: :: :: :::::

SpaC TTTGGTGACCCCAATAATCCGGATTATCAGATTCGAAAATATGCTAAAGAGACGAATACC

290 300 310 320 330 340

250 260 270 280 290 300

SpaC-c CCGGGCCTGTATGATGTGTACCTGAACGTTAAAGGTAATAAGCAGCAAAACGTTAAACCG

:: :: :::: :: :: :: ::::::: ::::: ::::: :::::::: :: :: ::

SpaC CCTGGATTGTACGACGTTTATTTGAACGTCAAAGGCAATAAACAGCAAAATGTGAAGCCT

350 360 370 380 390 400

310 320 330 340 350 360

SpaC-c GTCGACATCGTTCTGGTGGTGGATATGAGCGGTTCTATGGAAAGTAATCGTTGGGGTACC

:: :: :: :: : :: :: :::::: :: :: ::::: :: : ::::: ::

SpaC GTAGATATTGTCTTAGTTGTTGATATGTCTGGGTCAATGGAGTCAAACAGATGGGGCACG

410 420 430 440 450 460

370 380 390 400 410 420

SpaC-c AACCGTGCAGGTGCTGTCCGTACCGGTGTGAAAAATTTTCTGACGAGCATTCAGAACGCG

:: :: :: :::::::: ::::: :: :: :: ::::: :::: ::::: :::::

SpaC AATCGAGCTGGTGCTGTTCGTACTGGCGTTAAGAATTTCTTGACTTCTATTCAAAACGCC

470 480 490 500 510 520

430 440 450 460 470 480

SpaC-c GGCCTGGGTAACTATGTTAATGTCGGCCTGATCGGTTTCAGCTCTCCGGGCTATATCGGC

:: :::::::: :: :: ::::: :: : :: :: :: ::: :: ::::::::

SpaC GGTCTGGGTAATTACGTCAATGTTGGTTTAATTGGGTTTTCTAGTCCTGGTTATATCGGT

530 540 550 560 570 580

490 500 510 520 530 540

SpaC-c GGTAAATCAGGTTACATTTCGGTTAAACTGGGCAAGGCCGGTAATGCTAGCCAGCAACAG

:: ::::: ::::: ::: :: ::: : ::::: :: :::::::: :::::::::::

SpaC GGCAAATCGGGTTATATTAGTGTCAAATTAGGCAAAGCAGGTAATGCCAGCCAGCAACAA

590 600 610 620 630 640

550 560 570 580 590 600

SpaC-c GCAATTAACGGTGCTCTGTCTCCGCGTTTTCAGGGCGGTACCTACACGCAAATCGGCCTG

:: ::::: ::::: :: ::: : ::::: :: ::::: :: ::::: :: :: ::

SpaC GCGATTAATGGTGCATTGAGTCCAAGGTTTCAAGGGGGTACGTATACGCAGATTGGTTTG

650 660 670 680 690 700

610 620 630 640 650 660

SpaC-c CGTCAGGGTAGTGCGATGCTGAATGCCGACACCTCCGGCAACAAAAAGATGATGATTCTG

:: :: :: :: ::::::::::: :::::: ::::: ::::: ::::::::: ::

SpaC CGGCAAGGATCAGCCATGCTGAATGCGGACACCAGTGGCAATAAAAAAATGATGATTTTG

710 720 730 740 750 760

670 680 690 700 710 720

SpaC-c CTGACCGATGGTGTCCCGACGTTTTCAAATGAAGTGATTAACTCGGAATGGATCAATGGC

: :: ::::: :: ::::: ::::: :: :: ::::: :: :: :: ::::: :::::

SpaC TTAACTGATGGCGTGCCGACTTTTTCTAACGAGGTGATAAATTCAGAGTGGATAAATGGT

770 780 790 800 810 820

730 740 750 760 770

SpaC-c ACCCTGTATGGTACGAACTTCGGCAGTTCCCGC---GATGAACCGGGTAATACCGCACGT

:: ::::::: :: :: :: :: ::: :: :::::::: :: :: ::::::::

SpaC ACATTGTATGGCACTAATTTTGGA---TCCAGCAGAGATGAACCAGGGAACACCGCACGA

830 840 850 860 870 880

780 790 800 810 820 830

SpaC-c CTGCGCTGGCCGTACACGGACTCATCGGGCCATTATATCTACGATACCTGGCCGGCGACG

:: :: ::::: ::::: :: :: :: :::::::: :: ::::: ::::: :: ::

SpaC CTTCGATGGCCATACACCGATAGTTCAGGTCATTATATATATGATACTTGGCCAGCAACA

890 900 910 920 930 940

840 850 860 870 880 890

SpaC-c CTGGGTGAAGCGAAAATTGCCAAGGATAGTGGCAACGAAGTGCATGCACTGGGTATCCAG

::::::: :: :: :: :: ::::::::::: :: :: ::::: :: : :: :::::

SpaC TTGGGTGAGGCCAAGATAGCAAAGGATAGTGGTAATGAGGTGCACGCGTTAGGCATCCAA

950 960 970 980 990 1000

900 910 920 930 940 950

SpaC-c CTGGCTGATGACGATCACTATATGACCAAAGAAAAGATCCGTCAAAATATGCAGCTGATT

:::::::: ::::: ::::: ::::: :::::::: :: :: ::::: :::::::: :::

SpaC CTGGCTGACGACGACCACTACATGACGAAAGAAAAAATACGCCAAAACATGCAGCTTATT

1010 1020 1030 1040 1050 1060

960 970 980 990 1000 1010

SpaC-c ACGAACTCACCGGACCTGTATGAAGACGCGGATTCGGCGGATGCCGTTGAAGCGTACCTG

:: :: :::::::: : :: ::::: :: ::: :: ::::: ::::: :: :: ::

SpaC ACCAATTCACCGGATTTATACGAAGATGCTGATAGTGCCGATGCTGTTGAGGCTTATTTG

1070 1080 1090 1100 1110 1120

1020 1030 1040 1050 1060 1070

SpaC-c AACAATCAGGCCAAGGATATCATCAAGAACTTCAACACCGTGACGGACGGCACCATTACG

::::::::::: ::::: :: ::::: ::::: :: :: :: :: :::::::: :: ::

SpaC AACAATCAGGCAAAGGACATTATCAAAAACTTTAATACTGTCACCGACGGCACGATCACA

1130 1140 1150 1160 1170 1180

1080 1090 1100 1110 1120 1130

SpaC-c GATCCGATCGGTACCCAATTCCAGTATGCAAACAATCAGGCTACCGTTACGAGCGTCGGC

:: ::::: ::::: ::::: :: ::::: ::::: ::::: ::::::::::: ::::::

SpaC GACCCGATTGGTACGCAATTTCAATATGCGAACAACCAGGCGACCGTTACGAGTGTCGGC

1190 1200 1210 1220 1230 1240

1140 1150 1160 1170 1180 1190

SpaC-c AAACAGACCGTTCCGGCAAGCGAACTGCCGTCTGCAGCCATTCAAGACGGCCAGCTGACC

:: :: :: :: :: ::::: :: :::: ::: :: :: ::::: :: :: ::::

SpaC AAGCAAACTGTGCCAGCAAGTGAGTTGCCAAGTGCGGCGATCCAAGATGGTCAATTGACG

1250 1260 1270 1280 1290 1300

1200 1210 1220 1230 1240 1250

SpaC-c GTGAACCATATGAATCTGGGTCAAGATCAGGAAGTGCAAATCCACTATCAGGTTCGCATT

::::: :: ::::: ::::::: ::::::::::: :::::::: ::::: :: :: ::

SpaC GTGAATCACATGAACTTGGGTCAGGATCAGGAAGTTCAAATCCATTATCAAGTACGGATC

1310 1320 1330 1340 1350 1360

1260 1270 1280 1290 1300 1310

SpaC-c AAAACCGAAGACGCCGGTTTTAAGCCGGATTTCTGGTACCAGATGAACGGCGAAACCCTG

::::: :: :: :: :: :: ::::: ::::: :::::::: ::::: :: ::::: ::

SpaC AAAACAGAGGATGCTGGCTTCAAGCCTGATTTTTGGTACCAAATGAATGGTGAAACATTG

1370 1380 1390 1400 1410 1420

1320 1330 1340 1350 1360 1370

SpaC-c CTGACGCCGAAAGCAGGTGCAGCTGCGGTGGATTTTGGTATCCCGAGCGGTCGTGCACCG

:::: :: ::::: :: :: :: :: :: :: ::::: :: :: :: : :::::

SpaC TTGACACCAAAAGCGGGCGCTGCCGCTGTTGACTTTGGGATTCCTTCAGGCAGGGCACCA

1430 1440 1450 1460 1470 1480

1380 1390 1400 1410 1420 1430

SpaC-c GCTACCACGGTGTATGTTCAAAAACAGTGGCGTCAACTGAGCAATCAGTCTCTGCCGGAT

:: :: :: :: ::::: :: :: :: ::::: :: : :::::::: :: : ::::::

SpaC GCAACTACAGTTTATGTGCAGAAGCAATGGCGCCAGTTAAGCAATCAATCGTTACCGGAT

1490 1500 1510 1520 1530 1540

1440 1450 1460 1470 1480 1490

SpaC-c ACCCTGAACGTCACGGTGCAGCGTAAAGTTGCGGACGGCTCCCTGGATCCGAATTGGCAA

:: :: ::::::::::::::::: ::::: :: ::::: :: :: ::::: :::::::::

SpaC ACGCTCAACGTCACGGTGCAGCGAAAAGTGGCTGACGGTTCGCTTGATCCAAATTGGCAA

1550 1560 1570 1580 1590 1600

1500 1510 1520 1530 1540 1550

SpaC-c CAGACCCTGGTCCTGAAAAAGGCGGATAACTGGAAAGCCTCTTTTACCGCGCCGGCCTAT

:::::: : ::::: ::::: :: :::::::::::::: ::::: :: :: :: :::

SpaC CAGACCTTAGTCCTTAAAAAAGCTGATAACTGGAAAGCTAGCTTTACGGCACCTGCGTAT

1610 1620 1630 1640 1650 1660

1560 1570 1580 1590 1600 1610

SpaC-c AACAATCAAGGCCAGAGTTTCTCCTACGTCGTGAAAAGTGAAGACGCGTCCGGTATCGAT

:::::::: :: :: ::::: :: :: ::::: :: :::::::: :: :: :: :: :::

SpaC AACAATCAGGGTCAAAGTTTTTCATATGTCGTTAAGAGTGAAGATGCCTCGGGAATTGAT

1670 1680 1690 1700 1710 1720

1620 1630 1640 1650 1660 1670

SpaC-c CTGAGCTCTTTCATTAGTTCCCAGAATATGGATCAACAGACCGCCACGCTGACCCTGACG

:::: :: :: :: ::::: :: ::::::::::: :: :: :: ::: :::: ::::

SpaC TTGAGTTCGTTTATCAGTTCTCAAAATATGGATCAGCAAACAGCAACGTTGACTTTGACA

1730 1740 1750 1760 1770 1780

1680 1690 1700 1710 1720 1730

SpaC-c AACCAACAGTATGGCTTTCAATTCCAGAAAAAGACCACGGACGGTACCGATCTGAGCGCA

:: :: :::::::: ::::: :: :::::::: :: :: :: ::::: ::: : :::

SpaC AATCAGCAGTATGGTTTTCAGTTTCAGAAAAAAACAACCGATGGTACTGATTTATCAGCA

1790 1800 1810 1820 1830 1840

1740 1750 1760 1770 1780 1790

SpaC-c GACCAACTGAAAGCTATGCAGTTTAATCTGACCCAGTACAGTGATAACTCCTTCCAACAG

:: :: :::: :: ::::::::::: : ::::::::::: :::::: :: :: :::

SpaC GATCAGTTGAAGGCCATGCAGTTTAACTTAACCCAGTACAGCGATAACAGTTTTCAGCAG

1850 1860 1870 1880 1890 1900

1800 1810 1820 1830 1840 1850

SpaC-c GCGAGCAAGACGAATGCCATTACCTCTACGGATCTGCAGGCACTGGCTCCGGGCTATTAC

:: ::: :: :: ::::: :: :: ::::::::::::::::: :: :: :: ::::::

SpaC GCATCCAAAACCAACGCCATCACGTCAACGGATCTGCAGGCACTAGCGCCAGGGTATTAC

1910 1920 1930 1940 1950 1960

1860 1870 1880 1890 1900 1910

SpaC-c GGTATCCAGGAAGCCGCAGCTCCGACCGGCTATCAGCTGGATGGTACCACGTACCTGTTT

::::: :::::::: ::::: :: :: :: ::::: :: ::::: :: ::::: :: :::

SpaC GGTATTCAGGAAGCTGCAGCACCTACAGGTTATCAACTTGATGGGACAACGTATCTTTTT

1970 1980 1990 2000 2010 2020

1920 1930 1940 1950 1960 1970

SpaC-c CAGCTGACCAGCGACGGCCAATGGCAGTATCATGGTACCAAAGATAACGTTACGTCAGGC

::::: :: :: :: :::::::: :: ::::: :: :: :: :: :: :: :::::

SpaC CAGCTAACGTCTGATGGGCAATGGCAATACCATGGCACAAAGGACAATGTGACATCAGGG

2030 2040 2050 2060 2070 2080

1980 1990 2000 2010 2020 2030

SpaC-c TCGGTCATTAATGGTCAACAGACCCTGAACCCGGTGGGCGACAAATCTGACGATTTCACC

:: :::::::: :: ::::: :::: :: :: :: :: :: :: :: ::::: ::

SpaC AGTGTTATTAATGGCCAGCAGACTTTGAATCCTGTTGGTGATAAGTCAGATGATTTTACG

2090 2100 2110 2120 2130 2140

2040 2050 2060 2070 2080 2090

SpaC-c GTTACGGGTGATCACCAACAGATTCTGACCCTGACGAAATATGACGAACCGAAGCCGAGC

:: :: :: :::::::: :: :::::::: :: ::::::::::: ::::: ::::: :

SpaC GTGACCGGGGATCACCAGCAAATTCTGACGCTAACGAAATATGATGAACCAAAGCCATCC

2150 2160 2170 2180 2190 2200

2100 2110 2120 2130 2140 2150

SpaC-c ATGACCCTGCGCGTTATCAAACAGGATAATCAATCTCAGTACCTGGCAGGTGCAGCATTT

::::: :::: :: :::::::::::::::::: :: :: :: ::::::::::: ::

SpaC ATGACTTTGCGGGTCATCAAACAGGATAATCAAAGCCAATATCTTGCAGGTGCAGCGTTC

2210 2220 2230 2240 2250 2260

2160 2170 2180 2190 2200 2210

SpaC-c ACCCTGCAGCCGAGTGCAGGTGAAGCTGAAACCATTACGTCATCGGCAACCTCCGAGGGT

:::::::: :: ::::: :: :::::::: :: :: :: :::::::: :: :: :::::

SpaC ACCCTGCAACCAAGTGCTGGCGAAGCTGAGACGATAACATCATCGGCGACATCTGAGGGA

2270 2280 2290 2300 2310 2320

2220 2230 2240 2250 2260 2270

SpaC-c CAGGCATTCGCAACGAAACTGGTGGCAGACGGTACCTATACGATGAGTGAAACCAAGGCT

:: :: :: :: :: ::: : :: ::::: ::::::::::::::: ::::: :: ::

SpaC CAAGCGTTTGCGACAAAATTAGTTGCAGATGGTACCTATACGATGTCAGAAACAAAAGCA

2330 2340 2350 2360 2370 2380

2280 2290 2300 2310 2320 2330

SpaC-c CCGGATGGCTACCAGTCCAACCCGGCGAAAATTGCCATCCAGGTCGCAACCACGGGCAAG

:: :::::::: :: ::: :: :: :: :::::::: ::::: :: :: :: :: ::

SpaC CCAGATGGCTATCAAAGCAATCCTGCAAAGATTGCCATTCAGGTAGCTACGACTGGTAAA

2390 2400 2410 2420 2430 2440

2340 2350 2360 2370 2380 2390

SpaC-c GAAGCTACCGTGACGATTGATGGTGAAGCCCTGAAACCGGGCGAATCAAAGAATGGTTAT

:: :: ::::: :::::::: ::::: :: :::: ::::::::: ::::: :: ::

SpaC GAGGCAACCGTCACGATTGACGGTGAGGCATTGAAGCCGGGCGAAAGTAAGAACGGATAC

2450 2460 2470 2480 2490 2500

2400 2410 2420 2430 2440 2450

SpaC-c ACCCTGGCAATTGATGGCTCGACCATCACGCTGCAAGCGATTAACCAGCCGCTGGCAATC

:: : :: ::::::::: :: ::::: :::: :::::::: ::::: :: :::::

SpaC ACATTAGCGATTGATGGCAGCACGATCACTTTGCAGGCGATTAATCAGCCACTTGCAATT

2510 2520 2530 2540 2550 2560

2460 2470 2480 2490

SpaC-c CTGCCGCACACCGGCGGTCAAGGTTACCAGCGTCTGCT

::::::: :: :: ::::: :: :: ::::: ::::

SpaC TTGCCGCATACAGGTGGTCAGGGCTATCAGCGATTGCT

2570 2580 2590 2600
